# The anti-tumorigenic activity of A2M—A lesson from the naked mole-rat

**DOI:** 10.1371/journal.pone.0189514

**Published:** 2017-12-27

**Authors:** Susanne Kurz, René Thieme, Ronny Amberg, Marco Groth, Heinz-Georg Jahnke, Philipp Pieroh, Lars-Christian Horn, Marlen Kolb, Klaus Huse, Matthias Platzer, Daniela Volke, Faramarz Dehghani, Anton Buzdin, Kathrin Engel, Andrea Robitzki, Ralf Hoffmann, Ines Gockel, Gerd Birkenmeier

**Affiliations:** 1 Institute of Biochemistry, University of Leipzig, Medical Faculty, Leipzig, Germany; 2 Department of Visceral, Transplantation, Thoracic and Vascular Surgery, University of Leipzig, Medical Faculty, Leipzig, Germany; 3 Leibniz Institute on Aging—Fritz Lipmann Institute (FLI), Jena, Germany; 4 Centre for Biotechnology and Biomedicine, Molecular Biological-Biochemical Processing Technology, University of Leipzig, Germany; 5 Department of Anatomy and Cell Biology, Martin Luther University Halle-Wittenberg, Grosse Steinstrasse 52, Halle (Saale), Germany; 6 Institute of Pathology, Division of Breast, Gynaecological and Perinatal Pathology, University of Leipzig, Leipzig, Germany; 7 Center for Biotechnology and Biomedicine, University of Leipzig, Leipzig, Germany; 8 Pharmaceutical Artificial Intelligence Department, Insilico Medicine, Inc., Emerging Technology Centers, Johns Hopkins University at Eastern, B301, Baltimore, Maryland, United States of America; 9 Department of Pathway Engineering for Cancer Research, OmicsWay Corp., Walnut, CA, United States of America; 10 National Research Centre “Kurchatov Institute”, Centre for Convergence of Nano-, Bio-, Information and Cognitive Sciences and Technologies, 1, Akademika Kurchatova sq., Moscow, Russia; Universitat des Saarlandes, GERMANY

## Abstract

Cancer resistance is a major cause for longevity of the naked mole-rat. Recent liver transcriptome analysis in this animal compared to wild-derived mice revealed higher expression of alpha2-macroglobulin (A2M) and cell adhesion molecules, which contribute to the naked mole-rat’s cancer resistance. Notably, A2M is known to dramatically decrease with age in humans. We hypothesize that this might facilitate tumour development. Here we found that A2M modulates tumour cell adhesion, migration and growth by inhibition of tumour promoting signalling pathways, e.g. PI3K / AKT, SMAD and up-regulated *PTEN* via down-regulation of miR-21, *in vitro* and in tumour xenografts. A2M increases the expression of CD29 and CD44 but did not evoke EMT. Transcriptome analysis of A2M-treated tumour cells, xenografts and mouse liver demonstrated a multifaceted regulation of tumour promoting signalling pathways indicating a less tumorigenic environment mediated by A2M. By virtue of these multiple actions the naturally occurring A2M has strong potential as a novel therapeutic agent.

## Introduction

The naked mole-rat (NMR), (*Heterocephalus glaber*), a subterranean rodent, tolerates hypoxia, hypercapnia, avoids many physiological characteristics associated with aging and, most importantly, exhibits pronounced resistance to cancer [1, 2]. Transcriptome analysis of NMR liver compared to wild-derived mice revealed very high expression of cell adhesion molecules involved in tumour development as well as the pan-proteinase inhibitor alpha2-macroglobulin (A2M) [3]. Earlier, we have shown that the level of A2M in human blood decreases with age [4] and exposition of tumour cells with activated A2M (A2M*) inhibited many malignancy-associated properties of tumour cells *in vitro* by inhibition of members of the WNT/ß-catenin pathway [5]. Therefore, we hypothesized that the reduction of A2M in aged humans may facilitate tumour development. A2M is a pan-proteinase inhibitor in human blood and tissue capable to bind most proteinases and many growth factors, hormones and cytokines [6]. Binding to its receptor, the low density lipoprotein receptor-related protein 1 (LRP1, also known as CD91), mediates fast clearance of tethered peptides and proteins [7]. A specific role of A2M in cancer cell metabolism and development has not been elaborated in detail yet. Here we show that A2M* modulates tumour cell adhesion, migration and growth by inhibition of central signalling pathways such as phosphatidylinositol 3-kinase (PI3K), protein kinase B (AKT) and SMAD. A2M* up-regulates the tumour suppressor phosphatase and tensin homolog (*PTEN)*, *CD29 (ITGB1)* and *CD44* but does not evoke epithelial-mesenchymal-transition (EMT). Furthermore, A2M* was found to down-regulate microRNA-21 (miR-21), which is a dominant inhibitor of *PTEN* expression. Notably, A2M* inhibits growth of tumours in nude mice independent of their origin, and induces tumour necrosis in tumour tissue and tumour slices cultures. Transcriptome analysis displayed fundamental and unexpected insights in regulatory power of this ancient and highly conserved human plasma protein. The unique features of using A2M* as a novel anti-tumorigenic therapeutic in cancer patients prompted us to performing this study.

## Materials and methods

### Regulatory

Human heparin plasma was obtained from male healthy volunteers aged between 30 and 40 years. All participants provided their written informed consent to participate in this study. The local ethic committee of the Faculty of Medicine of the University of Leipzig, Germany, approved this study in accordance to the ICH-GCP guidelines (reference number: 057-2010-08032010). All animal studies were carried out in strict accordance with the recommendations for the care and use of animals and were approved by the”Regierungspräsidium Sachsen”, Germany (reference number: 24–9168.11/18/11).

### Purification and quality control of A2M

A2M was purified from human citrated blood obtained from healthy donors (Blood Donation Center, Leipzig) as described earlier [4]. The purity of the final product was proved by native pore-gradient polyacrylamide gel electrophoreses (PG-PAGE) (4–20%) (native PG-PAGE), Rate PAGE, or sodium dodecyl sulfate (SDS)-PG-PAGE (8%) under reducing or non-reducing condition. Transformation of A2M by methylamine and rate gel electrophoresis was performed as described earlier [56].

### Characterization of purified A2M

Human A2M purified from pooled plasma preserved its native structure and can be transformed to its active form, A2M*, by thiol ester disruption [58]. This induces a major conformational change as indicated by Rate PAGE, binding to immobilized LRP1 and the responsiveness to the receptor-associated protein (RAP) (*LRPAP1*) inhibition. The final product used for *in vitro* and *in vivo* stimulation experiments exhibited insignificant stimulatory effects on white blood cells indicating the absence of lipopolysaccharide (LPS), and factors accidentally co-purified with A2M could not be detected by mass spectroscopy (**S1 Fig**).

### Receptor binding of A2M

LRP1 (2 μg/mL) (#04–03, Biomac, Leipzig, Germany) was coated onto 96-well plates, followed by incubation with a standard solution of A2M/A2M* and the test samples. Bound A2M/A2M* was detected by Horseradish (HRP)-labelled polyclonal anti-A2M-imunoglobulin (Ig).

### Cell line and cell culture

Cell lines used for this study are prostate cancer cell line PC-3 (ATCC, CRL-1435), astrocytoma cell line 1321N1 (ECACC, 86030102), lung cancer cell line A549 (ATCC, CCL-185™), breast cancer cell line MDA-MB 231 (ATCC, HTB-26), colon rectal cancer cell line HT29 (ATCC, HTB38), and melanoma cell line LOX (DCTD TUMOR REPOSITORY). Cells were cultured at a density of 10^6^ /mL in RPMI 1640 medium, containing penicillin (100 U/mL), streptomycin (100 μg/mL), glutamine (2 mM) and 10% fetal calf serum (FCS). Cells were seeded 48h and the medium was replenished 24h prior adding of A2M*. In case of IGF-1 and TGFß1 studies, FCS was omitted after cells had settled. Cultures were incubated in a moist atmosphere with 5% CO_2_ at 37°C.

### Preparation of cell lysate

Cytosolic and nuclear cell extracts were prepared according to the Abcam guidelines (ww.abcam.com). All lysis buffer contained 0.3% protease inhibitor cocktail (P-8340) and 2 mM phenylmethylsulfonyl fluoride (PMSF), 1 mM sodium orthovanadate, 10 mM sodium fluoride (SigmaAldrich, Taufkirchen, Germany) in case of phosphorylation detection. Determination of protein concentration was performed according to Bradford [59].

### Immunoblotting

Isolated proteins (5–10 μg) and cell/tissue lysate (20–200 μg) were subjected to SDS-PG-PAGE (4–20%), homogeneous SDS-PAGE (8%) under reducing or non-reducing conditions or to native PG-PAGE (4–20%). Proteins were blotted to nitrocellulose membranes (Schleicher & Schuell BioScience GmbH, Dassel, Germany), detected by specific antibodies in conjunction with HRP-labelled goat anti-mouse/anti-rabbit-Ig. Band visualization was performed by chemiluminescence detection and densitometric analyses were performed by means of the E.A.S.Y. Win32 software (Herolab, Wiesloch, Germany). To determine the amount of immunoreactive protein all values were normalized to the control. Dilution of primary/secondary antibodies are tabulated in **(**S6 Table).

### Immunoprecipitation

Tumour cells were lysed in lysis buffer (0.025 M Tris, 0.15 M NaCl, 1 mM EDTA, 1% Nonidet P-40 and 5% glycerol, pH 7.4) freshly supplemented with 1 μg/mL aprotinin, 1 μg/mL leupeptin, 1 mM PMSF, 1 mM NaF and 1 mM Na3VO4. After centrifugation (13,000*×*g, 20min, 4°C), the supernatant was incubated with appropriated amount of Protein A-agarose for 1h at 4°C to reduce non-specific binding. Then, the supernatant was incubated with respective monoclonal antibodies (20 μg/mL) overnight at 4°C. Protein A-agarose was again added and incubated under shaking for 2h. The agarose beads were washed with PBS-T (10 mM sodium phosphate, 150 mM NaCl, 0.05% Tween-20) and finally subjected to SDS-PG-PAGE followed by Western blotting.

### Cell viability and proliferation assays

Trypan blue exclusion test was performed to evaluate cell viability. Cell proliferation was assayed in 96-well plates (5,000 cells/well) using the WST-1 assay (Sigma-Aldrich, Taufkirchen, Germany).

### Wound scratch assay

1321N1 astrocytoma cells were seeded in petri dishes and cultured until complete adherence and confluence. Cell monolayers were scratch with a p10 pipet tip, and fresh medium was added containing increasing concentration of A2M* (0, 10, 50, 100 nM), and culturing was continued. The distance of gap closure was measured under phase-contrast microscope daily. Each experiment was performed 3 times with 8 replicates for each treatment. The data were expressed as mean ± s.d.; Student *t*-test was employed.

### Adhesion assay

5,000 cells were seeded into 6-well plates and cultured in the absence or presence of A2M* or RAP (10, 30, 100 nM) at 37°C, 5% CO_2_ for 24h. Cell detachment was accomplished by 0.05% trypsin for 3 min. Remainder cells were fixed with para-formaldehyde (4%) and stained with Gentiana violet followed by intensive washing to remove excess dye. Finally, cells were treated with acetic acid and the liberated dye was measured spectrophotometrically at 590 nm. The absorbance represents the number cells left attached to the bottom of the well (*n* = 3) (*P < 0.*05*, **P < 0.*01*).

### Tumor xenografts

Subcutaneous tumor xenografts were generated in 8 weeks old male athymic nude mice (Crl:NU-Foxn1nu, Charles River Laboratories, Sulzfeld, Germany). The animals were kept in a 12h day and 12h night cycle and had unrestricted access to drinking water and food. Briefly, 10^6^ tumor cells (A549, 1321N1 or PC-3) were resuspended in 100μL RPMI-1640 medium and 100μL matrigel (BD Biosciences, Heidelberg, Germany), before injected subcutaneously. Therefore the mice were anaesthetized and analgesia was done by ketamine and xylazine 10 minutes before the tumor cell injection. Tumors were grown for 7 days and treated with 5.6 mg A2M* *i*.*p*. three times per week for 31 days. Animals were sacrificed by neck dislocation after CO_2_/O_2_ anesthesia. Tumors were then removed, weighted and fixed either in 4% formalin for histology and immunohistochemistry or were shock frozen in liquid nitrogen for molecular analyses.

### Whole blood assay

Whole blood assay was applied to trace for bacterial by-products in the A2M preparations as described earlier [60]. Three different A2M preparations were tested at a final concentration of 0.8 μM that corresponds approximately to 50% of the concentration of a bolus injection into a mouse (5.6 mg/20 g body weight).

### Impedance spectroscopy and proteomics

Impedance spectroscopy was performed in 2D and 3D cell cultures using Multiwell-Multiel-Ectrode-Arrays (MMEA) [61] and proteomics was conducted by means of nanoRP-HPLC-QqTOF-MS/MS by measuring tryptic peptides. Detailed data are presented in **(S1 Protocol)**.

### Flow cytometry

Adhered A549 cells were cultured in the presence of various concentrations of A2M* at 5% CO_2_ and 37°C for 15h. Cells were washed with PBS and treated by trypsin/EDTA for dissolution. After washing with PBS cells were immune-stained with mouse anti-human CD44-PE. Intracellular labelling of CD44 was performed using the BD Cytofix/Cytoperm Plus fixation/permeabilization kit according to the manufacturer’s protocol. The PE labelled isotype IgG1 antibody was used for control **(S6 Table)**.

### Histochemistry

After sacrificing the animals, tumours were either fixed by 4% (v/v) paraformaldehyde buffered in 0.2 M phosphate-buffer, pH 7.0, for at least 24h, or tumour slice cultures (TSC) (350 μm) were prepared immediately using a vibratome. The slices were cultured, A2M* was added and incubation was continued for 48h followed by fixation. Prepared paraffin-embedded tissue sections (4 μm) were stained for HE, dehydrated and mounted on a microscope slide with Entellan. Ki67 nuclear labelling index was calculated as the percentage of Ki-67 immunoreactive nuclei divided by the total number of analysed tumour cells. Vascular and blood cells were excluded. Tumour cells were counted in five representative areas of the sections at a magnification of x 400. Between 500 and 2000 cells were counted per section from each specimen. Used primary and secondary antibodies are presented in **(S1 Protocol)**.

### Analyses of gene expression

For the analysis of gene expression, total RNA was isolated using the peqGold total-RNA kit (VWR, Darmstadt, Germany). Isolation was performed according to manufacturers’ instruction. RNA integrity (clear visibility of 28S and 18S ribosomal RNA bands) of total RNA was assessed by running an aliquot on a 1.5% agarose gel stained with ethidium bromide. For miR-21 analysis standard Trizol RNA isolation protocol was used. Reverse transcription was done using Revert Aid first stand kit (Life Technologies, cat. K1622) with random hexamer primers. For mature miR-21 the stem loop primer was used. Target genes were amplified pipetting 25 ng cDNA, 0.5 μM of each primer, 100 μM dNTPs, 1 x GoTaq buffer ready to load and 0,025U/μl GoTaq polymerase (Promega). PCR (3 step) was performed using the PTC 200 Multicycler (Biozym). Amplicons were analysed and visualized on 1.5% agarose gels under an ultraviolet transilluminator. For quantitative PCR SYBR-Green Mix (Roche) was used. Amplification and analysis was done using the Stratagene MX3000p and a standard SYBR green amplification program (annealing 60°C, 45 cycles) with melting curve. Specific Primers are listed in **(S7 Table)**.

### Transcriptome analysis of A549 cells

A549 cell cultures, both control and treatment (30 nM A2M*, 24h) were pelleted and suspended using 50 μl RNase-free water. RNA isolation was performed using the QIAamp RNA Blood Mini Kit, according to manufacturer specifications. The resulting RNA was quality controlled using Qubit RNA HS Assay and NanoDrop 2000c. Library preparation was done using ScriptSeq™ Complete Gold Kit with the required additional FailSafe™ PCR Enzyme Mix following the Epicentre® manual. The library preparation was executed on a Caliper Life Sciences Sciclone G3, (Perkin Elmer). To validate the quality of the cDNA library, we performed both a Qubit™ (Qubit dsDNA HS Assay) and LabChip GX® (DNA 1K / 12K / Hi Sensitivity Assay LabChip and DNA High Sensitivity Reagent Kit) measurement. We used Angencourt RNAClean XP and Agencourt AMPure XP beads in the respective washing steps.

The library quality was checked by sequencing the multiplexed libraries on a MiSeq using a MiSeq Reagent Kit v2 (300 cycle) in 2x150 cycles mode. The afore prepared pool was then sequenced in two runs on a NextSeq 500 using a NextSeq® 500/550 High Output Kit v2 (300 cycles) in 2x150 cycles mode. The NextSeq was controlled by NextSeq Control Software v1.4. Reads were extracted into FastQ files using Illumina’s BCL2FastQ v2.16.0.10, on default setting, arriving at an average of 54 million reads per sample (ranging from 49 to 67 million).

For each sample (six with A2M*treatment and fives PBS controls), reads were aligned to the Ensembl Genome GRCh38.p2 Release 80 using a two-step approach. Firstly, reads were mapped with default parameters with the splice-aware alignment program Hisat v2.0.0b [62]. Secondly, reads, for which no alignment position was reported by Hisat, were then mapped with Bowtie2 v.2.2.6 [63, 64]. The abundance of transcript isoforms (TPM–transcripts per million[65]) was determined using StringTie v1.1.2 [66]. Expression values were extracted from the appropriate StringTie gtf output files and Welch’s unequal variances t-test was performed on transcript level in order to identify differentially expressed transcripts. The resulting set of transcripts was filtered for a nominal p-value less to 0.05 and for a measurable expression value (greater zero) in both the control and experimental group. Transcripts with a measurable expression value in only one of the two groups were either labelled as switched off (expression value of zero in treatment group) or switched on (expression value of zero in control group).

Finally, for calculating the phenotype correlation of the A2M* treatment and the PBScontrol group against the lung adenocarcinoma stages of the Cancer RNASeq Nexus [35], we calculated the average expression value for each transcript in each group. To compensate for the different count metrics (TPM or FPKM) used in the Cancer RNASeq Nexus, depending on their source data set, we calculated the averages for both TPM and FPKM, as StringTie provides both metrics. The transcript expression averages were then correlated against the various stages I through IV of the lung adenocarcinoma samples as well as the adjacent normal. Each transcript was weighted with *w*_*i*_ according to their p-values *p*_*i*_ provided by the Cancer RNASeq Nexus to distinguish between each stage and the adjacent normal, using the following formula.

$$w_{i}=\frac{\left|\log_{10}p_{i}\right|}{\sum_{i}\left|\log_{10}p_{i}\right|}$$

### Transcriptome analysis of liver and xenografts in mice

Total RNA was extracted using the prepGold total RNA kit. Isolation was performed according to manufacturer’s instruction. Library preparation was done as described in [67]. Deviating from that in brief, the RNA integrity number (RIN) varies between 8.1 and 9.2. Around 1 μg of total RNA was used for library preparation using Illumina TruSeq RNA sample prep kit v2 following the manufacturer's description. Sequencing was done on a HiSeq2500 using cluster generation and sequencing chemistry v3 (TruSeq SR Cluster Kit v3—cBot—HS; TruSeq SBS Kit v3—HS). Reads were extracted in FastQ format using Illumina’s BCL2FastQ v1.8.4 v1.8.4 allowing one mismatch in the index sequence. The sequencing resulted in around 48 million reads per sample.

For separating the mice and human tissue derived reads in the xenografted samples they were introduced into the tool xenome [68]. As graft (human) and host (mouse) reference sequence in the xenome tool a transcriptome of the according species was used. The transcriptome datasets were prepared using TopHat v2 setting parameter—transcriptome-index in combination with–G option. The (a) human transcriptome was prepared based on the human reference genome hg19 and RefSeq annotation from 2014-11-07 and the (b) mouse transcriptome on the mouse reference genome mm10 and the RefSeq annotation from 2014-06-08. After classification the batch of reads assigned to the human reference was used for further analysis. These reads were mapped accordingly [69] using TopHat v2 using the human reference sequence as mentioned above.

Reads obtained from liver samples were also mapped using TopHat v2 using the murine reference sequence as listed above.

Counting of reads per gene was done based on the resulting sam files using the tool featureCounts [70] and the gene annotation as mentioned above. Count data were loaded into GNU R for further analysis. In order to check the relation of the samples to each other for both datasets (liver and tumour), a heat map was created based on the Spearman correlation coefficient using the count data. The counts were normalized to RPKMs (reads per kilobase transcript and million mappable reads) [71] using the exon lengths given by featureCounts. To identify DEGs the packages edgeR [72] and DESeq [73] were used. Genes are regarded as differentially expressed if the p-value of both packages is ≤ 0.01.

### Differential expression analyses and pathway analyses

Differential pathway analysis was performed with OncoFinder [46]. In order to comply with the previously established protocol [64], raw reads were filtered by quality > 30 score through FASTX toolkit and then trimmed at 5‘ and 3‘ in order to remove index and adapter. Only the remaining reads were used for alignment with TopHat v2 [69] against the human genome assembly (GRCh37). After determination of gene expression levels [74] the reads were normalized using Bioconductor DESeq2 tool [75], resulting RPKM in values. The signalling pathways knowledge base developed by SABiosciences (http://www.sabiosciences.com/pathwaycentral.php) was used to determine structures of intracellular pathways, which were used for OncoFinder, as described previously [76]. For the functional annotation of the primary gene expression data, we applied our original algorithm OncoFinder [46]. It enables calculation of the Pathway Activation Strength (PAS), a value which serves as a qualitative measure of pathway activation. Briefly, the enclosing algorithm utilizes the following formula to evaluate pathway activation:

$$PAS_{p}=\sum_{n}ARR_{np}\cdot BTIF_{n}\cdot \mathrm{lg}\left(CNR_{n}\right)$$

### Statistical analyses

Unless stated otherwise, student’s two-tailed t-test was used to compare differences between 2 groups. One- or two-way ANOVA were used to analyze differences between more than 2 groups. All data are presented as means s.e.m. using GraphPad Prism 6.0 (GraphPad Software, Inc., La Jolla, CA). *P* < 0.05 was considered to be significant. The numbers of animals and individual samples used are described in the appropiate figure legend.

## Results

### A2M* affects cell adhesion, migration and cell contacts

**Fig 1:**
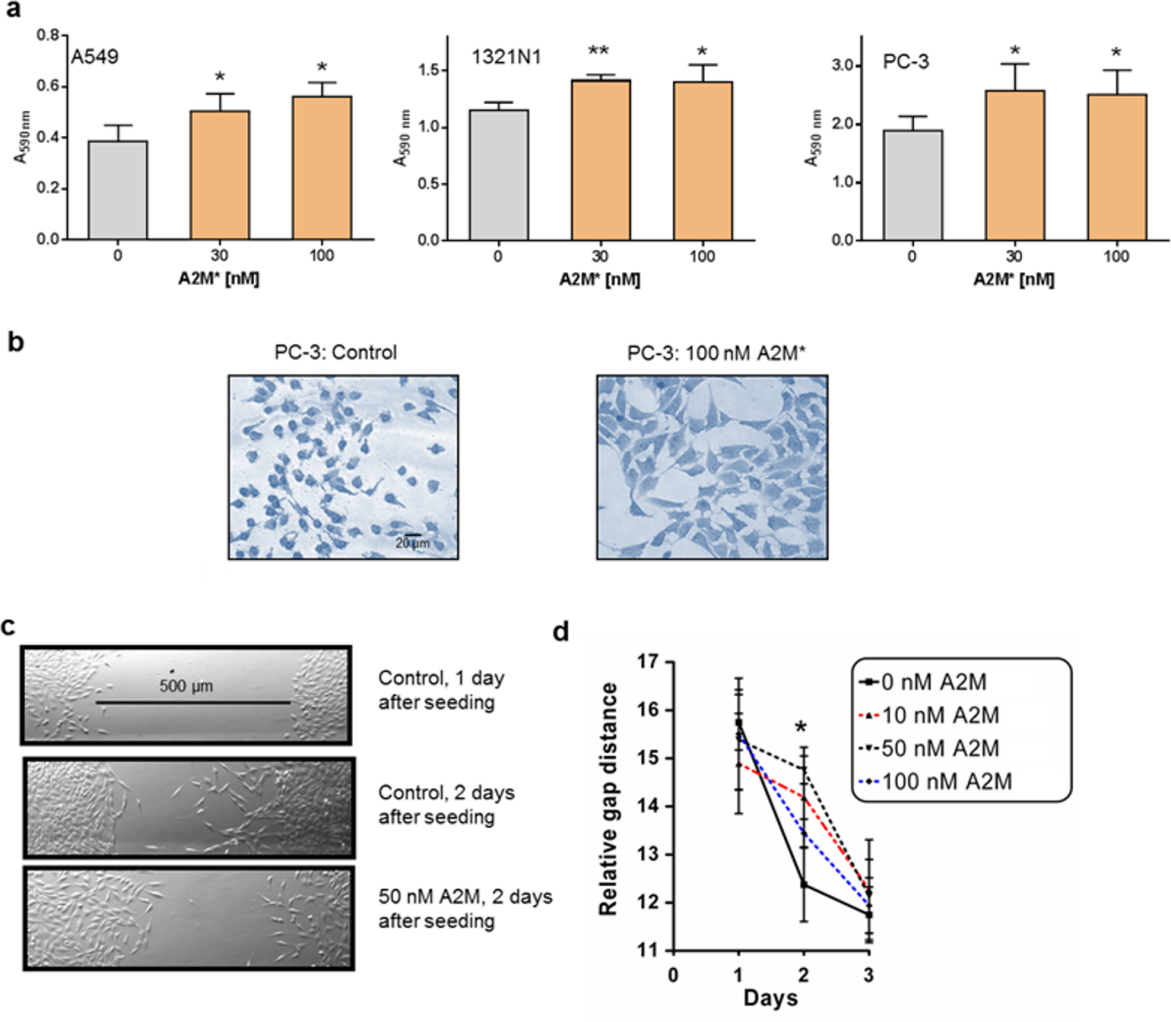
Effect of A2M* on adhesion and migration of tumour cells. **(a)** Effect of A2M* on adhesion of the tumour cell lines A549, 1321N1 and PC-3 (*n* = 3; mean ± s.d, *t*-test). **(b)** Light microscopy of attached cells of non-treated and A2M-treated PC3 cell. **(c-d)** Scratch assay of 1321N1 cells treated with A2M* at different concentration (0, 10, 50, 100 nM) and analysis of gap closure time (*n* = 3). Error bars mean ± s.d, *t*-test) (*P < 0.05, **P < 0.01).

A2M* is a ligand of LRP1, which has been shown to regulate cytoskeleton organization, interaction with the extracellular matrix and affects migration and invasion of various non-tumour and tumour cells [8, 9]. Specifically, LRP1 appeared to promote maturation and endocytosis of ß1-integrin (CD29) [9] and may regulate the surface expression of CD44 [10]. Accordingly, we could show that A2M* promotes adhesion of different tumour cells (A549, 1321N1 and PC-3) at nanomolar concentrations **(Fig 1A and 1B)**. The observed increase in cell adhesion might be causative of the inhibition of cell motility as analysed by the wound scratch assay **(Fig 1C and 1D)**.

**Fig 2:**
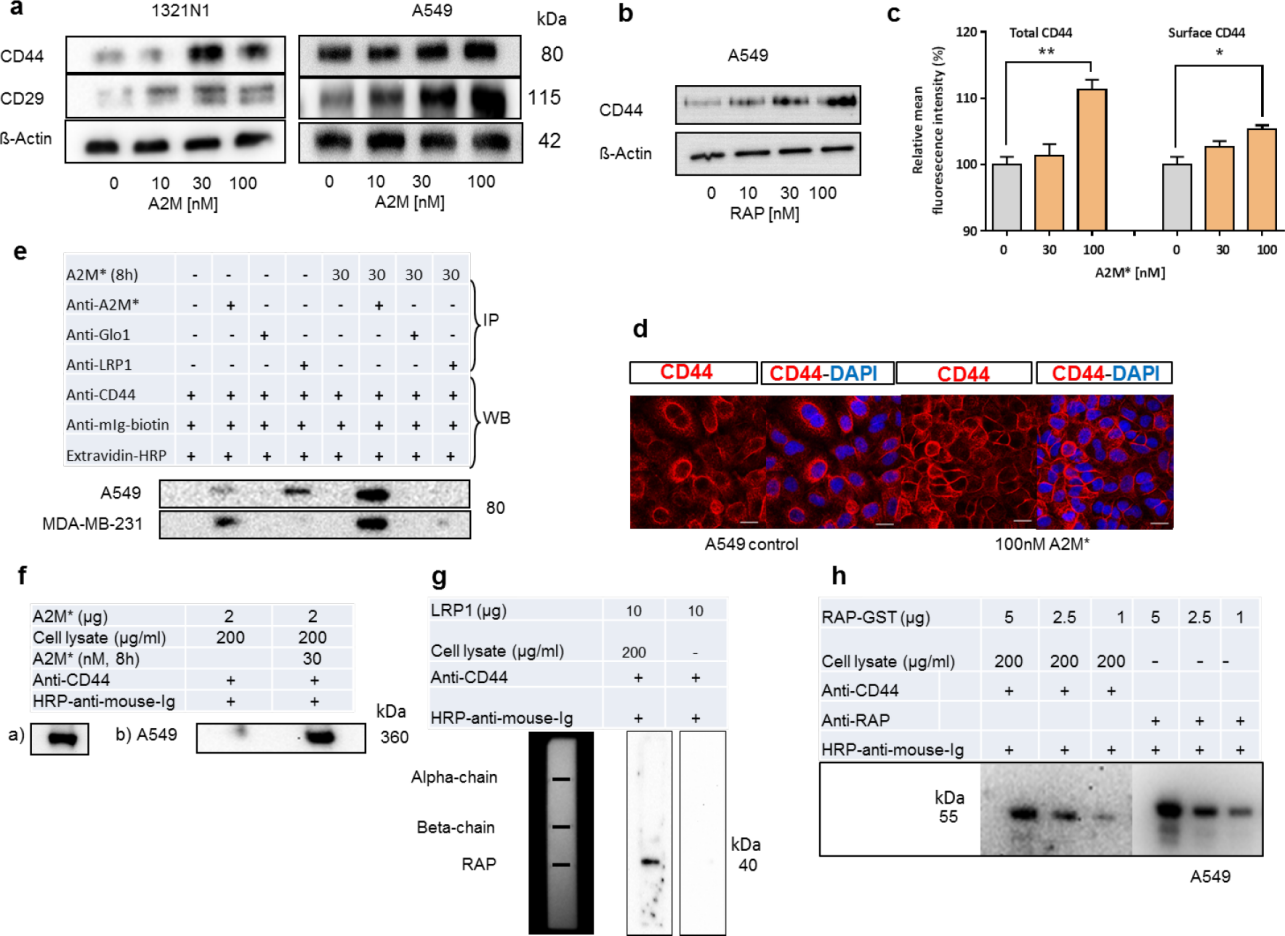
Effect of A2M* on expression of CD44 and CD29 in different tumour cell lines. **(a)** 1321N1 and A549 cells were cultured in the absence and presence of increasing concentrations of A2M, lysed and immunoblotted for CD44 and CD29. **(b)** A549 cells were stimulated by RAP and treated as shown in (a). **(c)** A549 cells were treated with A2M* and expression of total and cell surface CD44 was analysed by flow cytometry (n = 3), error bars mean ± sem, t-test (*P < 0.05, **P < 0.01). **(d)** A549 cell were treated with 100 nM A2M* for 12h, fixed by paraformaldehyde and immunestained for CD44 and counterstained with DAPI. **(e)** Immunoprecipitation of CD44 in A2M*-stimulated and non-stimulated A549 and MDA-MB-231 cells. Cells were treated with 30 nM A2M* for 24h, lysates were precipitated with different antibodies (anti-LRP1, anti-A2M*, anti-GLO1) and Western blotted for CD44 protein. **(f)** Ligand blot of A2M* for CD44 binding. A2M* was blotted to membranes which were incubated with cell lysate of A549 afore stimulated by 30 nM A2M* for 8h. Binding of CD44 to immobilized A2M* was detected by anti-CD44/HRP-anti-mouse-Ig. a) Immobilized A2M* detected by anti-A2M*/HRP-anti-mouse-Ig and b) Detection of binding of CD44 to immobilized A2M*. **(g)** Ligand blot demonstrating binding of CD44 to LRP1-associated RAP. Membrane-blotted LRP1 was incubated with A549 cell lysate and bound CD44 was detected by respective antibodies. **(h)** Recombinant RAP was electro-blotted, followed by incubation with A549 cell lysate. Detection of CD44 bound to immobilized RAP was accomplished by anti-CD44/HRP-anti-mouse-Ig. Immobilized RAP was detected by anti-RAP antibody (blotting control).

To examine the contribution of membrane adhesion molecules, we analysed the A2M* dependent expression of *CD29* and *CD44* in 1321N1 and A549 cells **(Fig 2)**. A2M* increased the expression of both *CD29* and *CD44* in 1321N1 cells and A549 cells **(Fig 2A)** similarly to the receptor associated protein (*RAP*) **(Fig 2B)** in A549 cells. The latter effect was previously reported for thyroid carcinoma cells and supports the assumption of a tight interaction between the hyaluronan receptor CD44 with LRP1 [10]. The authors assumed that stimulation of cell attachment might be caused by inhibition of receptor-mediated endocytosis by RAP leading to surface accumulation of CD44. As A2M* stimulates rather than inhibits LRP1 turnover [7], we looked for an alternative explanation. RT-PCR analysis revealed that A2M* was unable to induce a *CD44*-isoform shift, which is essential for cells undergoing EMT [11], *in vitro* and *in vivo* and to modulate *CD44* transcript level in A549 cells. Because, A549 cells showed a stronger increase in CD44 and CD29 increase after A2M* treatment, FACS analysis of these cells demonstrated an enhanced total cellular as well as plasma membrane-anchored CD44 upon A2M* treatment **(Fig 2C)**. Similar results were obtained by immunocytochemistry indicating accumulation of CD44 immunoreactivity at adjacent cell membranes **(Fig 2D)**. These data indicate that A2M* might regulate CD44 expression at a translational or post-translational rather than transcriptional level.

Treatment of A549 cells with 30 nM A2M* followed by precipitation of cell lysates by an A2M*-specific monoclonal antibody (mab) showed co-precipitation of cellular CD44 that was not observed in the presence of the control antibody (anti-GLO1 mab) of the same isotype **(Fig 2E)**. Less immunostaining for CD44 was seen in non-treated cells indicating that the CD44 co-precipitation signal may depend on A2M* levels. In accordance with the recent literature [10], immunoprecipitation by a mab directed against the ß-chain of LRP1 revealed co-precipitation with CD44, but only in cells that were not stimulated with A2M*. This encouraged our assumption that CD44 might in addition to LRP1 interact with A2M*. This was further proved by ligand blotting showing that immobilized A2M* extracted CD44 from cell lysate **(Fig 2F)**. Using purified LRP1, we were able to perform ligand blots with A549 cell lysates, which surprisingly disclosed the interaction of CD44 with RAP rather than with subunits of LRP1 **(Fig 2G)**. Similar results were obtained by ligand binding studies using recombinant RAP **(Fig 2H)**.

This is in contrast to the work of Perrot et al. [10], who found that CD44 is bound to a truncated receptor comprised by the ligand-binding cluster IV. As RAP binding is mapped to all four LRP1 clusters, we assume that CD44-LRP1 interaction is mediated through tightly bound receptor-associated RAP. Another possibility of the CD44-LRP1 is an interaction bridged by A2M*, which is known to ligate receptor domain II and IV similar to CD44 [10]. The fact that RAP is mainly present in the cytosolic compartment and only few molecules appear associated to LRP1 at the outer cell surface, it is reasonable to assume that A2M* plays a dominant role in regulation of CD44 expression in tumour cells compared to RAP.

Here we found that A2M* increases the expression of *CD29* and *CD44* in different tumour cell lines **(Fig 2A)**. Integrins such as CD29 (ß1-integrin) bind to the extracellular matrix (ECM) and thus connect the cytoskeleton to ECM, which is accompanied by signal transduction [12]. Adhesion of cells is integrin-dependent as *CD29* knockdown has been shown to reduce cell adherence [13]. Recently, LRP1 has been shown to interact with *CD29* and regulates its surface expression [9]. Whether A2M* acts together with LRP1 as an additional component to steer also the expression of *CD29* has to be elucidated.

**Fig 3:**
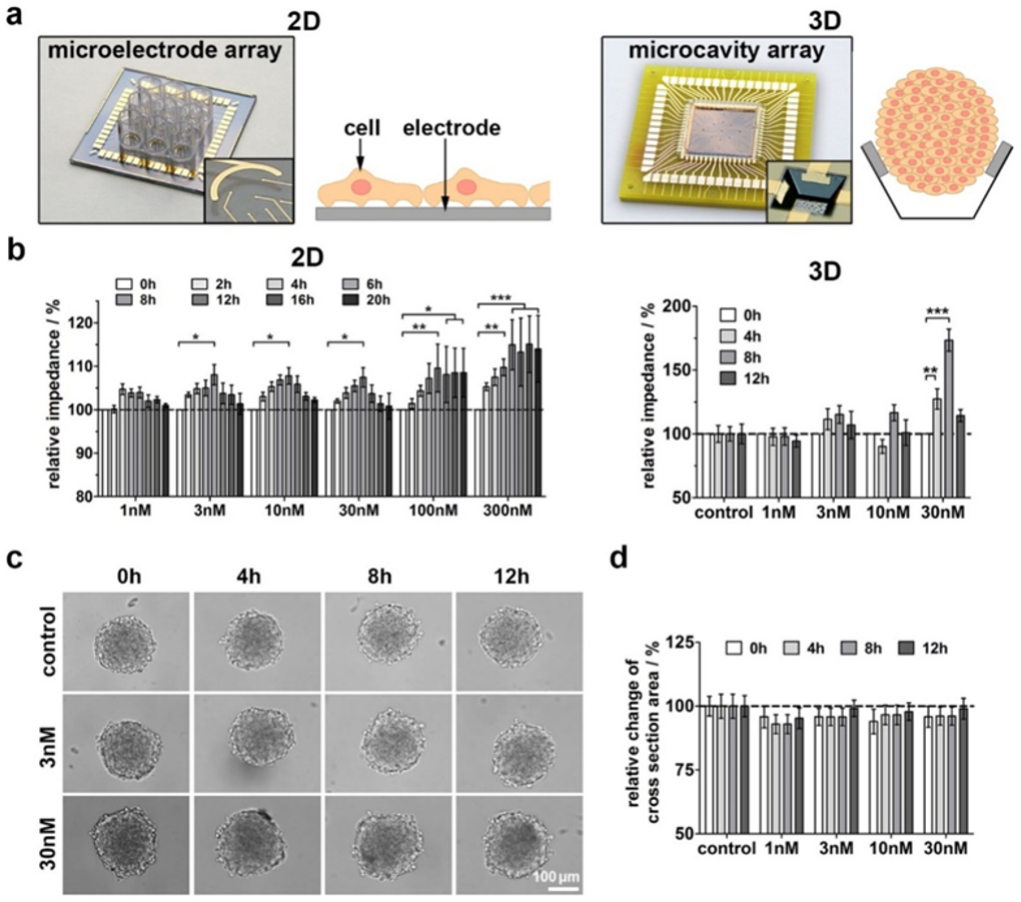
Impedance monitoring of A2M* effects on 2D and 3D cultures of 1321N1 cells. **(a)** Microelectrode- and microcavity arrays that were used for monitoring 2D and 3D cultures. **(b)** Impedimetric analysis for selected time points after application of different concentrations of A2M* to 1321N1 cells in 2D cultures (*n* = 5) and 3D cultures (*n* = 10 spheroids). **(c)** Microscopic images of impedimetrically analysed spheroids upon treatment with A2M* at different time points. **(d)** Statistical analysis of A2M* effect on the spheroid size, measured as cross section area over experiment time (*n* = 10 spheroids). All values were normalized to time point zero as well as the control group and are presented as mean ± sem (*P < 0.05, ** *P* < 0.01, ***P < 0.001).

Recently, we investigated the impact of A2M and LRP1 on cellular properties of 1321N1 astrocytoma cells [5]. Therefore, we performed the impedance spectroscopy based monitoring of cell-cell interactions in 2D- and 3D-cell cultures by using microelectrode arrays with an electrode diameter of 100 μm for 2D cultures and microcavity arrays with a cavity edge length of 200 μm for 3D cultures in these cells **(Fig 3A)**. After seeding 1321N1 cells on collagen-coated microelectrode arrays A2M* (1 to 300 nM) was applied and impedimetrically monitored for up to 20h. The statistical analysis of selected time **(Fig 3B)** revealed a continuous increase of relative impedance even at the lowest concentration of 3 nM A2M* up to 8h. Afterwards, this effect diminished for concentrations up to 30 nM A2M*. Cellular alterations that could reflect this impedimetric effect course could be among others an increased cell-cell and/or cell substrate adhesion [14]. This correlates with the increased adhesion of tumour cells upon A2M* treatment **(Fig 1A)**.

In 3D-cultures of single spheroids (d = 150 μm) A2M* treatment significantly increased the impedance at 4h and 8h and 30 nM A2M*, respectively, but disappeared after 12h **(Fig 3B)**. Additionally, microscopic images were taken from each spheroid at each time point **(Fig 3C)**, showing no morphological alterations. Furthermore, the spheroid size was statistically analysed by determination of the cross section area **(Fig 3D)**. In comparison to the control group, a slight but not significant reduction of spheroid size was observed (3–7% at 8h). As nonspecific effects of A2M* like proliferation on cell size in 3D cultures could be excluded, there is strong evidence for an A2M*-induced transient strengthening of cell-cell contacts in a small time window of 4 to 12h (S1 Protocol).

### A2M* failed to induce EMT

**Fig 4:**
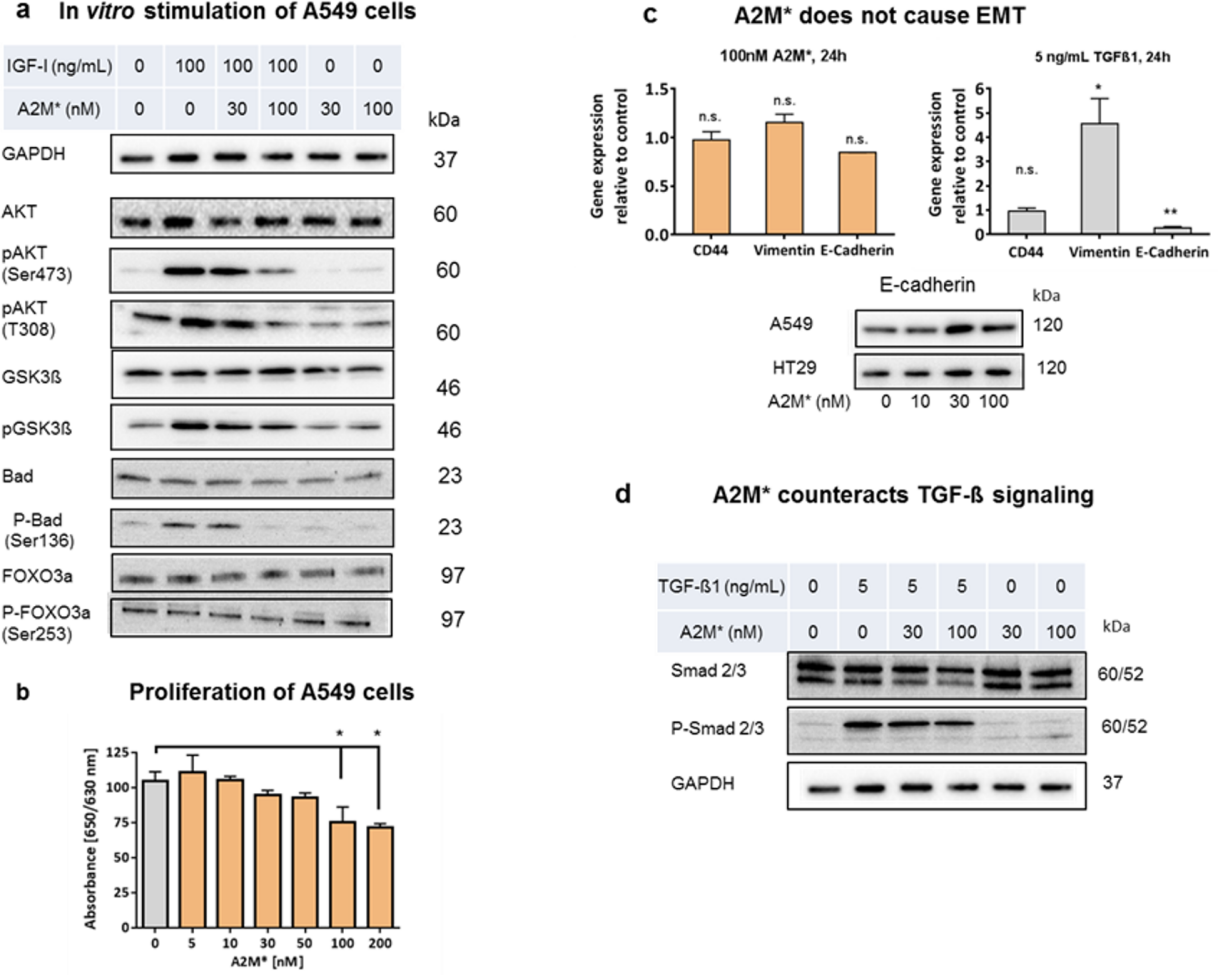
Impact of A2M* at signalling pathway members and EMT in tumour cells. **(a)** A549 cells were cultured in the absence (control) or presence of IGF-1 (100 nM) and/or A2M* (30 and 100 nM) for 8h. Cells lysates were analysed by immunoblotting for detection of non-phosphorylated and phosphorylated proteins using antibodies as in shown in **(S6 Table)**. **(b)** Inhibition of proliferation of A549 cells by A2M* analysed by WST-1 (*n* = 3), error bars are mean ± sem, *t*-test. **(c)** A2M* does not induce epithelial-mesenchymal transition (EMT). A549 cells were cultured in the presence of A2M* (100 nM) or TGF-ß1 (5 nM) for 24h followed by gene expression analysis by qRT-PCR of *CD44*, vimentin and E-cadherin (*n* = 4), error bars are mean ± sem, *t*-test). Immunoblot of E-cadherin in A549 and HT29 cells stimulated with A2M* (0, 10, 30, 100 nM) for 24h normalized to GAPDH. **(d)** A2M* counteracts TGF-ß1 signalling in A549 cells by inhibition of SMAD phosphorylation. All experiments were performed in triplicates. (*P < 0.05, **P < 0.01).

High expression of *CD44* was found to correlate with EMT, which describes the transition of cells from an epithelial to a mesenchymal phenotype that plays a crucial role in tumour proliferation, invasion, and metastasis. EMT promotes expression of *CD44* that is under control of several regulatory factors such as *TWIST*, *SNAIL* (*SNAI1*), *ZEB1* and *SLUG* (S*NAI2*). In contrast, the epithelial cadherin (E-cadherin), an epithelial marker, participates in the negative regulation of EMT [15]. It is well known that insulin-like growth factor 1 (IGF-1) [16] and transforming growth factor ß1 (TGF-ß1) [17] are strong inducers of EMT. Both are known to bind A2M tightly [18]. For this reason we studied the effect of A2M* on growth factor-stimulated tumour cells. IGF1 is known to stimulate the PI3K/AKT pathway as well as the RAS/MEK/ERK cascade in cancer cells, which will finally end up in enhanced cell growth and inhibition of apoptosis [12]. Targeting the IGF-1 axis has already been shown promising anti-tumour effects in the clinics [19]. To analyse the role of A2M* to EMT, we conducted these experiments with A549 cells, as they showed an increase in CD44 cell surface expression after A2M* treatment. As seen, A2M* abated the effect of IGF-1 on AKT kinase pathway by preventing AKT phosphorylation and downstream inactivation of GSK3B (**Fig 4A**). This finally could explain the inhibition of tumour cell proliferation shown in **Fig 4B**. In addition, A2M* inhibited also phosphorylation of BAD (BCL2-associated death promotor). Phosphorylation of BAD by AKT at Ser112 or Ser136 normally results in the loss of its pro-apoptotic activity [20]. Non-phosphorylated BAD induces apoptosis by inhibiting anti-apoptotic BCL2 family members, thereby activating the apoptotic effector machinery. Therefore, the suppression of BAD phosphorylation by A2M* seems to be a promising target to induce apoptosis in cancer cells.

The EMT inducing property of TGF-ß1 is confirmed by increased RNA expression of the mesenchymal marker vimentin and decrease expression of E-cadherin, while no effect was observed on the expression of *CD44* **(Fig 4C)**. In contrast, A2M* displayed no significant effect on transcription level of CD44, vimentin and E-cadherin indicating that A2M* is not inducing EMT. This is all the more important as A2M* increased the cellular E-cadherin protein level in A549 and HT29 cells **(Fig 4C)** and antagonized the TGF-ß1 induced phosphorylation of SMAD2/3 transcription factors **(Fig 4D)**.

### A2M* reduced tumour growth in nude mice

**Fig 5:**
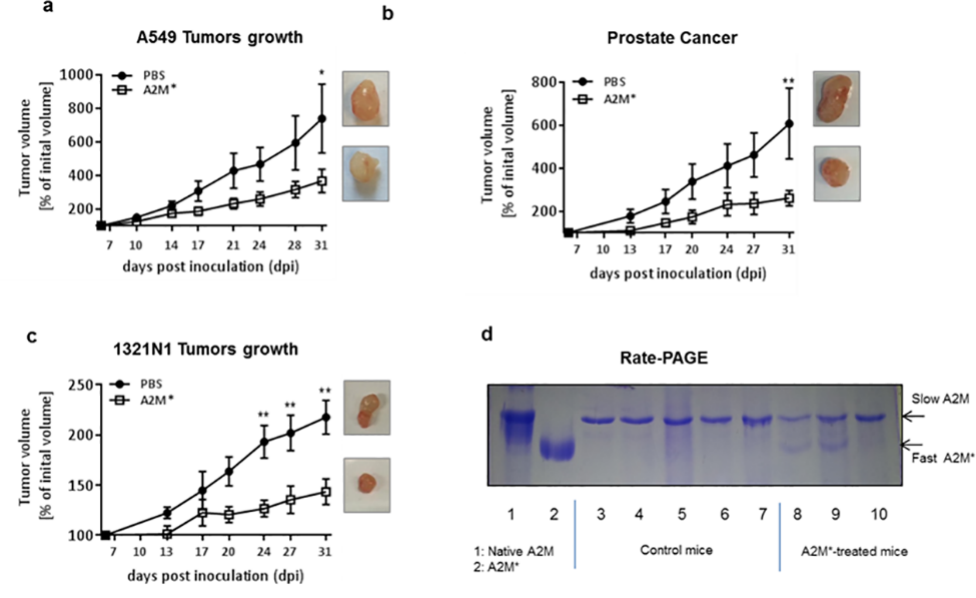
A2M* decreases growth of tumour xenografts in mice. Nude mice were subcutaneously injected with 10^6^ tumour cells at day 1. At day 7 mice were treated with 5.6 mg / 20 g body weight A2M* (*n* = 10) *i*.*p*. and control mice (*n* = 10) obtained PBS. Injection was repeated every second day and was accompanied by calibration of the tumour size. The experiments were stopped at day 31 by euthanizing the animals. **(a)** A549 lung carcinoma cells, **(b)** PC-3 prostate carcinoma cells and **(c)** 1321N1 astrocytoma cells. Error bars represent mean ± sem (2-way ANOVA test with Bonfererroni’s multiple comparison correction). **(d)** Analysis of mice plasma for the presence of injected A2M*. Plasma of control mice (lanes 3–7) and A2M*-treated mice (lanes 8–10) were subjected to Rate-PAGE. Purified native (slow-migrating) and transformed A2M* (fast-migrating) are shown in lane 1 and 2, respectively. (* *P* < 0.05, **P < 0.01).

To reveal *in vivo* effects of A2M*, nude mice were inoculated with 3 different cancer cell lines subcutaneously and treated intraperitoneally (*i*.*p*.) with A2M* *(*5.6 mg/20g body weight). A2M* treated mice showed a reduced growth of A549, 1321N1 and PC-3 tumours compared to control groups (*n* = 10) **(Fig 5A–5C)**. Injected human A2M* could be detected in the blood of animals as indicated by the presence of the fast-moving A2M* form in treated mice **(Fig 5D)**.

A2M* did not affect the animals’ body weight, but caused alterations of some blood cell parameters such as an increase of neutrophils and elevation of the monocyte chemotactic protein 1 (MCP1, also known as CCL2) level in isolated spleen cells of A2M*-treated animals **(S2A–S2C Fig)**. This chemokine is known to recruit monocytes, dendritic and memory cells to sites of injury [21].

In contrast to controls, tumours of A2M*-treated animals showed a shrunken capsule together with multiple necrosis in different stages together with damaged tumour cells. The overall picture of the tumour was dominated by infiltrating macrophages in areas of tumour cell degeneration and at tumour margins **(S3 Fig)**. The high accumulation of tumour-infiltrating macrophages in A2M*-treated mice may arise from induction of MCP1 by A2M* as shown above.

Tumour tissues slice cultures (TSC) (350 μm) were prepared to eliminate the influence of remote organs on tumour behaviour in A2M*-treated animals. In control TSCs, the tumours were bordered by a fibrous capsule composed of tight connective tissue showing only few cells presenting signs of mitosis or apoptosis **(S4 Fig)**. A2M*-treated TSCs showed a decline in the number of tumour cells with large areas of scar formation at all A2M* concentrations investigated. Immunostaining for activated caspase-3 showed only few cells within the tumour with a random distribution pattern. Staining for Ki-67 was higher in control TSCs than in A2M*-treated slices.

### A2M* modulates the expression of PTEN and miR-21

**Fig 6:**
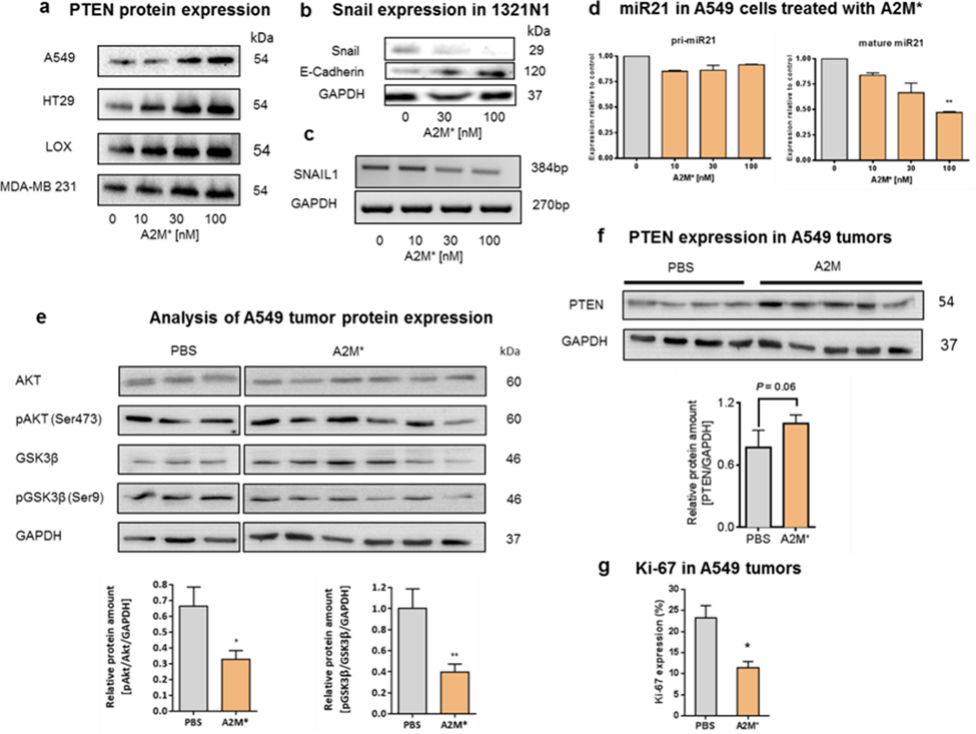
Effect of A2M* on expression of PTEN and miR-21 *in vitro and in vivo*. **(a)** Various tumour cells (A549, HT29, LOX, MDA-MB 231) were cultured in the presence of A2M* (0, 10, 30, 100 nM) and cell lysates were probed for PTEN by immunoblotting. Protein amounts loaded were normalized to GAPDH levels, **(b)** 1321N1 cells mutated for *PTEN* were treated with A2M* and expression of *SNAIL* and E-Cadherin compared to GAPDH was studied by immunoblotting, **(c)** Separation of PCR products of *SNAIL* and *GAPDH* in 1.5% agarose gels **(d)** A549 cell were cultured in the presence of A2M* (0, 10, 30, 100 nM) for 8h followed by qRT-PCR analysis of pri-miR-21 and mature miR-21 transcripts (*n* = 3), error bars are mean ± sem. **(e)** A549 tumours from mice were homogenized and analysed by immunoblotting for AKT/pAKT, GSK3B/pGSK3B, GAPDH and followed by semi-quantitative pixel analysis (*n* = 3). Error bars are mean ± sem (*t*-test), **(f)** Analysis of PTEN expression in A549 tumours. Data are means ± sem of *n* = 2 technical replicates. **(g)** Tumour slices obtained from A2M*-treated and non-treated mice were immunostained for Ki-67 and quantitatively analysed. Data represent mean ± sem (*n* = 5; *t*-test) (*P < 0.05, **P < 0.01).

PTEN is a negative regulator of the intracellular levels of phosphatidylinositol-3,4,5-trisphosphate. Loss of this tumour suppressor gene is implicated in breast cancer progression and resistance to targeted therapies, and is thought to promote tumorigenesis by activating PI3K signalling [22]. Restoring *PTEN* function is sufficient to down-regulate both PI3K and MAPK signalling and triggers dramatic tumour regression [23]. Here, we found that A2M* treatment of A549, HT29, LOX and MDA-MB231 cancer cells caused up-regulation of the PTEN protein **(Fig 6A)**. Particularly in 1321N1 astrocytoma cells, where *PTEN* expression is absent due to a mutation [24], we found down-regulation of SNAIL at protein **(Fig 6B)** and mRNA level **(Fig 6C)** and expectedly an increase of the expression of E-cadherin [25]. To elucidate responsible upstream mechanisms, we checked for miR-21, known to promote tumour proliferation and invasion in many cancer types by targeting PTEN [26]. We observed suppression of mature miR-21 by A2M* without affecting the pri*-*miR-21 level **(Fig 6D)**. MiR-21 is overexpressed in almost all solid tumours, where it is involved in the genesis and progression of human cancer [27] and tumour environment modulation via SNAIL [25]. Thus, A2M* interfered with activity of one of the most frequently aberrant miR and its up-regulation has been reported to directly targeting many tumour suppressors in human cancers. So far A2M* effectively curbs the miR-21*/*PTEN/AKT signalling cascade.

Analogously, we found significant inhibition of AKT *(P* < 0.05) and GSK3B (*P* < 0.01) phosphorylation in tumour xenografts of A2M* treated mice **(Fig 6E)**. Analysis of *PTEN* expression revealed an increase of PTEN protein level by A2M* treatment although not reaching significance (*P* = 0.06) **(Fig 6F)**. These findings were in accordance with a decline in tumour cell proliferation measured by the number of Ki-67 positively stained nuclei **(Fig 6G)**.

### Positive feed-back regulation of A2M

A2M* is rapidly removed from circulation by endocytosis through LRP1 with a half-life time (T_1/2_) of 4 min [28], which represents one of the fastest clearance mechanisms in the human body. A2M is mainly synthesized in the liver, which is also the venue of its clearance due to abundant expression of LRP1 on hepatocytes [29]. This forced us to study the regulation of its expression in the liver of tumour-bearing mice, Balb/c mice and in cultured primary mouse hepatocytes. We found that exogenous A2M* enlivened the endogenous A2M synthesis in the liver of xenografts and Balb/c mice at mRNA- and protein level, which peaked 24h after injection (*n* = 3; *P* < 0.001) **(S5A–S5D Fig)** In addition, the endogenous expression of liver *A2M* mRNA was inducible by zinc orotate (*n* = 3; *P* < 0.01) **(S5E Fig)**. We have re-enacted the animal studies *in vitro* using primary mouse hepatocytes and found that only transformed A2M* but not native A2M is able to up-regulate its own expression by positive feed-back activation **(S5F Fig)**.

These findings raised the basic question, whether high concentration of mouse A2M on its own might be effectually enough for provoking anti-tumour effects. Therefore, we treated A549-xenotransplant bearing mice with zinc orotate (0.5 mg/g body weight) every second day. No effect in tumour growth was observed indicating that zinc substitution *per se* has no antitumor activity in the chosen concentrations, which correspond to the advised dosage for humans based on mg/kg body weight. (data not shown).

### Transcriptome analysis in A2M*-treated cells and tumor xenografts

**Fig 7:**
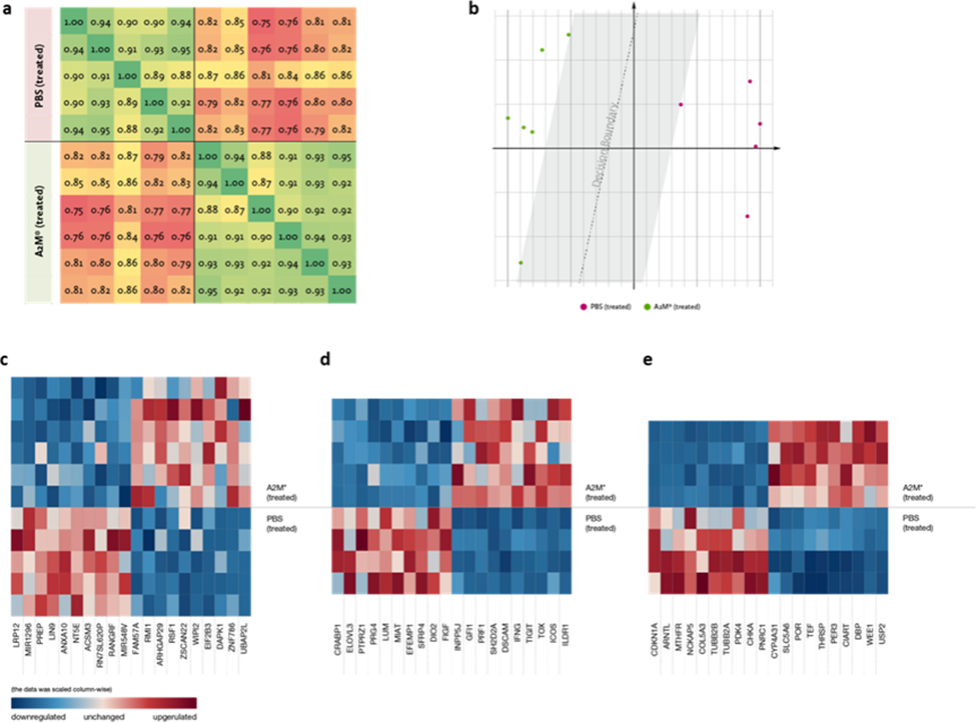
Heat maps of top transcripts modulated by A2M* measured by RNA-seq analysis. The **(a)** Pearson Correlation Matrix for all A549 samples using the transcripts with a P-value ≤ 0.05. The matrix resembles the underlying experiment with a r = 0.914 and a Spearman Rank Correlation of 0.978. **(b)** A MDS was performed, based upon all transcripts with a P-value ≤ 0.05. A clear decision boundary is visible between both groups, without any ambiguity in potential classification. The Scree Plot (not shown) states the trade-off point for potential classification in two dimensions. **(c)** Effect of A2M* on A549 tumour for the top ten down- and up-regulated transcripts with a P-value of less than 0.01. **(d)** Top ten down- and up-regulated genes with a P-value of less than 0.01 in xenograft stroma samples in A2M*-treated mice. **(e)** Top ten down- and up-regulated genes with a P-value of less than 0.01 in livers samples of A2M* treated mice. See also S1–S5 Tables.

The impact of A2M* (30 nM, 24h) on the transcriptome of cultured A549 cells was analysed by RNA-seq **(GEO GSE106261)** for completion of the in vitro analyses of A2M* treatment. Enrichment analysis (nominal *P < 0*.*01*) showed 55 DETs (Differentially Expressed Transcripts) down- and 74 up-regulated. Among those, the top five up-regulated DETs are transcripts of *UBAP2L*, *ZNF786*, *DAPK1*, *EIF2B3* and *WIPI2*. The top five down-regulated transcripts belong to *LRP12*, *miR-1296*, *PREP*, *LIN9* and *NED1* **(Fig 7A–7C)**. DETs from genes such as *RMI1* (up) and *NDE1*, *LIN9*, *ANXA10* (down) involved in mitosis and cell cycle are regulated in a manner consistent with the induction of cell cycle arrest. Modulation of transcripts of genes such as *ZPR1* (down) and *THTPA* (up) support the anti-proliferative effects of A2M*. Apoptosis related genes such as transcripts of *DAPK1* (up) and *NT5E* (down), matched with the histochemical findings showing increased amounts of apoptotic cells in A2M* treated tissue slices and tumor xenografts. Obviously, the down-regulation of miR-1296 in A2M*-treated cells may not follow the overall anti-tumour activity of A2M* as this miR is known to up-regulate oncogene *MCM2* [30]. However, PTEN associates with MCM2 and induces its de-phosphorylation and thus restricts replication fork progression. As we observed increased expression of *PTEN* in most tumours, that might override the effect of A2M* at miR-1296. An interesting aspect concerns the modulation of transcripts of *EIF2B3* and *PREP*. Both are involved in longevity and aging [31]. Malfunction of the initiation factor *EIF2B3* are known to make cells less tolerant to endoplasmic reticulum stress [31], and high cellular activity of the propyl endopeptidase (PREP) has been hypothesized to lead to an aggregation of α-synuclein, thus being a contributing factor for neurodegenerative diseases [31]. The significant up-regulation of *EIF2B3* and down-regulation of *PREP* in samples treated with A2M* might probably explain some of the anti-aging effects observed in naked-mole rats [1]. Furthermore, we found up-regulation of *ARHGAP29* that belongs to the Rho GTPase activating proteins (ARHGAP) which are involved in brain development [32], modulation of circadian genes [33] and show potential tumour suppressor activity in mantle cell lymphoma [34].

Overall, the correlation of the A2M*-treated samples is on average 9.8% higher than the correlation of the control samples when comparing against the adjacent normal transcriptome of each Stage-vs-Normal pair of the TCGA lung adenocarcinoma samples of Cancer RNASeq Nexus [35], and on average 1.4% lower when comparing against the various cancer stages of each. This indicates a small but apparent change in gene regulation in the A2M* treated cells towards normal lung tissue.

Due to the strongest effects of A2M* at growth of PC-3 xenografts **(Fig 5B)**, we decided to perform transcriptome analysis of tumors from these xenografts **(GEO GSE106989)**. Because no significant changes in PC-3 tumour tissues (adjusted *P < 0*.*01*) were observed, we decided to discriminate between human tumour PC-3 cells and mouse stroma. Considering the mouse specific reads, using the tool xenome for separation, we could identify 216 DEGs in A2M* treated mice compared to controls (*FDR < 0*.*01*), of which 156 were up- and 60 were down-regulated **(Fig 7D)**. Up-regulated genes primarily clustered in an induced immune-phenotype, being associated with the induction of the innate immune system. The top five fold-change wise up-regulated DEGs are *ILDR1*, *ICOS*, *TOX*, *TIGIT*, *IFNG*. This coincides with the occurrence of masses of macrophages in PC-3 xenograft slices and the up-regulation of *IFNG [36]*. The most abundantly down-regulated DEGs in the tumour stroma cells are more heterogeneous. The top five fold-change wise were *CRABP1*, *ELOVL3*, *PTPRZ1*, *PRG4 and LUM*.

As systemic A2M is mainly synthesized in the liver, we analysed how A2M* regulates the liver transcriptome of tumour mice **(GEO GSE107195)**. We identified 189 DEGs (*FDR < 0*.*01*) of which 86 were higher and 103 were lower expressed in A2M*treated mice liver compared to controls. The most fold-change wise up- and down-regulated liver DEGs were *USP2* and *CDKN1A*, respectively **(Fig 7E)**. USP2 is associated with the clock proteins in the liver [37]. On the other hand the senescence gene and cell cycle arrest inducer *p21* (cyclin dependent kinase inhibitor 1A, *CDKN1A*) is down-regulated in the liver of A2M*-treated animals. The *p21* is a negative prognostic marker in human hepatocellular carcinoma suggesting a role for A2M* in liver regeneration and cancer risk control [38]. Other significantly up-regulated genes in livers of A2M*-treated animals are *WEE1*, which blocks *CDK1* and *CIART*, *PER2* and *PER3* involved in the circadian rhythm [39, 40]. Modulation of the CLOCK-BMAL1-complex by PER2 is shown to inhibit HCV replication [41] and induces an oxygen-efficient glycolysis [42]. Collectively, these data reveal for the first time profound effects of A2M* on the circadian clock genes. The circadian rhythm and clock genes play a role in vaccination efficiency [43] and treatment regimens for several diseases [44]. The sensitivity of chemotherapeutics was linked with the functionality of the CLOCK/BMAL1 complex [45]. Implementation of defined treatment regimens or the usage of circadian rhythm influencing agents, like A2M*, could be a promising design for novel therapeutic applications.

Additionally, we built pathway activation profiles [46] for 388 intracellular signalling pathways that were tightly associated with cancer in previous works. The pathway activation strength (PAS) data clearly suggest that the A2M*-treated and untreated samples separate into two distinct groups with 189 significantly altered (104 up- and 85 down-regulated) (*P <* 0.02*)* characteristic molecular pathways.

## Discussion

A2M showed an extensive history as a protease inhibitor, immune modulator, and scavenger of growth factors, hormones and cytokines [10]. Here, we disclosed clear effects of A2M* on tumour cells *in vitro* and *in vivo*. Due to its fast endocytosis mediated by LRP1, it comprises one of the most important clearance systems of the extracellular space. Above all, it is involved in cell adhesion by modulation of the expression of cell surface molecules at protein and transcript level. Unexpectedly, A2M* interfered with one of the most important tumour suppressor, PTEN and its upstream modulator miR-21. A2M* was found to affect important genes not only of the tumour stroma but perturbs also important cell signalling pathways such as AKT that regulates diverse cellular processes including cell proliferation and survival, tissue invasion and angiogenesis. Many oncoproteins and tumour suppressors converge with AKT signalling transduction in an equilibrium that is altered in many human cancers by activating and inactivating mechanisms. Our results suggest that A2M* may guard this equilibrium by intervening at several cellular levels simultaneously. This is supported by exploring the effect on liver genes showing the up-regulation of branches of the AKT and RAS signalling pathways promoting cell survival and cell cycle progression, which may be considered as compensatory pro-survival mechanism, which is in contrast to its effect on tumour cells.

For the first time we disclosed the implication of A2M* in the regulation of circadian clock genes [47]. The mechanisms responsible for the connection between the circadian clock and cancer are not well defined, but disruption of circadian rhythms has been linked to tumorigenesis [48]. These findings may have a high impact for cancer chronotherapy as *BMAL1* (*ARNTL*) was recently characterized as tumour suppressor that increases sensitivity of cancer cells to chemical drugs **(Graphical abstract)** [49].

The overall observed strong tendency to induce cell cycle arrest in A2M*-treated cells together with the regulation of circadian clock genes in liver of A2M*-treated mice is an indicator for the known coupling of the two oscillating systems internal clock and cell cycle [50].

All data indicate that A2M fulfils important regulatory functions at the cellular level and among organs. As we showed earlier the blood level of A2M decreases significantly with age [4]. Assuming the proposed protective role of this protein in humans it might become clear now that a progressive A2M deficiency may abet tumour development especially under the view that most tumours develop in slow motion over a time period of about 10–20 years [51]. Ironically, the long-lived and cancer resistant naked-mole rats have remarkable higher level of A2M in blood and liver compared to humans and mice [3, 52]. We speculate that this might be an important reason for the outstanding cancer-resistance and lifespan of these animals.

Here we found that the degree of supplementation with zinc might obviously monitor the A2M level in blood. This is all the more important as A2M is the main zinc transporter in blood [53] delivering it to high-affinity binding proteins such as metallothioneins (MT) acting as intracellular zinc storage. Around 3,000 proteins are thought to bind zinc ions, which correspond to approximately 10% of the human proteome including masses of transcription factors [54]. The presence of metal-response elements (MRE) in the *A2M* gene promotor may explain the effect of external zinc supplementation on its expression [55].

## Conclusion

Our study strongly accentuates the requirement of activated A2M* for inhibition of tumour growth. In humans, native A2M represents approximately 99% of total *A2M* in the blood and only minute amount forms complexes with proteases (A2M*) in tissue or blood [56]. As A2M is sensing the proteolytic load in blood and tissue it provides a powerful regulator to coordinate the proteolytic/anti-proteolytic machinery and signalling pathway activation/inhibition. Thus, increasing the fraction of activated A2M* in humans might represent a novel approach for cancer prophylaxis and treatment. In this respect, the use of autologous A2M has already been considered in the clinics [57].

## Supporting information

S1 Fig Purification and quality control of A2M. **(a)** A2M was purified from human plasma and separated by native PAGE (4–20). **(b)** Purified native and transformed A2M (A2M*) were separated by Rate-PAGE into native (slow-migrating) and transformed (fast-migrating) A2M. **(c)** Three purified A2M preparations were separated by SDS-PAGE. The A2M* band was subjected to trypsin digestion and analysed by mass spectrometry to confirm the presence of A2M and to identify further co-migrating proteins. **(d)** Evidence for receptor binding of A2M*. LRP1 was coated to 96-well plates and incubated with increasing concentrations of A2M and A2M*. Bound A2M/A2M* was detected by HRP-labelled polyclonal anti-A2M-Ig. **(e)** Receptor-associated protein (RAP) (0–50 nM) inhibits binding of A2M* (10 nM) to immobilized LRP1. **(f)** Analysis of three A2M preparations for their stimulatory effect on human blood monocytes using the whole blood assay. Heparinized blood was incubated with medium (control), 10 ng/mL LPS and three purified A2M samples (A2M1, A2M2, A2M3), **respectively, at 5% CO_2_**, 37°C for 8h. Cells were centrifuged and the supernatant was analysed for TNF-alpha using cytometric bead array (CBA) (*n* = 3). Alb = albumin; Trf = transferrin, A2M = native A2M, A2M* = transformed A2M, RAP = receptor-associated protein.
(DOCX)

S2 Fig Analysis of blood cells in tumour-bearing mice before and after treatment with A2M*. (a) Coarse of body weight of tumour-bearing A549 mice treated with A2M* (n = 10) compared to control (n = 9). (b) EDTA blood was withdrawn from A549 tumour bearing mice and analysed in a ScilVet apparatus (ScilVet Animal Care Company, Viernheim, Germany). Blood cells were counted at day 7 after tumour induction (control) and day 31 after A2M* treatment. WBC–white blood cells, RBC–red blood cells, HGB—Hemoglobin, HCT–Hematocrit value, MCV–mean corpuscular volume, MCH–mean corpuscular hematocrit, PLT—platelets, MPV–mean platelet volume, RDW–red cell distribution width, LYM–Lymphocytes, MO—Monocytes, GRA—Granulocytes, (n = 9), (* P < 0.05, **P < 0.01, ***P < 0.001). (c), Effect of A2M* on mouse spleen cells. Spleen cells from A549 tumour-bearing mice treated with A2M* were isolated, stimulated with 10 nM lipopolysaccharide (LPS) or PBS (control) and cytokines were measured by cytokine bead arrays (CBA). (n = 10) (**P < 0.01). Error bars represent mean ± s.d.
(DOCX)

S3 Fig Morphological analysis of tumour tissue. Hematoxilin-eosin (HE) stained A549 tumour slices obtained from PBS-treated animals (control, a-d) and A2M*-treated animals (e-h). **(a)** Peripheral compartment of PBS treated tumour in overview. **(b)** Compact tumour organization with a few cells yielding apoptotic signs. **(c)** Tumour cells in a small area of tumour destruction (+) and cells with signs of apoptosis (arrow). **(d)** Dispersed vital A549 cells with few cells showing signs of degradation. **(e)** Peripheral compartment of an A2M*-treated tumour in overview. **(f)** Necrotic area (*) with macrophage accumulation the tumour tissue (arrow). **(g)** Low number of vital tumour cells paralleled by massive loss of tumour cytoarchitecture. **(h)** Loss of tumour tissue (*) accompanied by accumulation of macrophages (arrow). Scale bar: 300 μm **(a and e)**, 100 μm **(b-d, f-h)**.
(DOCX)

S4 Fig Morphological analysis of tumour tissue. Hematoxilin-eosin (HE) stained A549 tumour slices obtained from PBS-treated animals (control, a-d) and A2M*-treated animals (e-h). **(a)** Peripheral compartment of PBS treated tumour in overview. **(b)** Compact tumour organization with a few cells yielding apoptotic signs. **(c)** Tumour cells in a small area of tumour destruction (+) and cells with signs of apoptosis (arrow). **(d)** Dispersed vital A549 cells with few cells showing signs of degradation. **(e)** Peripheral compartment of an A2M*-treated tumour in overview. **(f)** Necrotic area (*) with macrophage accumulation the tumour tissue (arrow). **(g)** Low number of vital tumour cells paralleled by massive loss of tumour cytoarchitecture. **(h)** Loss of tumour tissue (*) accompanied by accumulation of macrophages (arrow). Scale bar: 300 μm **(a and e)**, 100 μm **(b-d, f-h)**.
(DOCX)

S5 Fig Effect of A2M* on expression of endogenous mouse A2M in the liver of A549-xenografted mice, Balb/c mice and isolated hepatocytes. **(a-c)** Liver of scarified mice were homogenized and analysed for A2M protein content and RNA by qRT-PCR and Western blotting. **(d)** Balb/c mice were injected with A2M* (5.6 mg/20g body weight), sacrificed after indicated times and the expression of mice A2M in the liver was analysed by qRT-PCR (*n* = 3 for each time point). **(e)** Balb/c mice were given a bolus injection of zinc orotate (0.5 mg/kg) (SigmaAldrich), and mouse *A2M* gene expression in the liver was determined by qRT-PCR. **(f)** Primary murine hepatocyte cultures from Balb/c mice were stimulated with native and transformed human A2M* (0–100 nM) for 24h followed by qRT-PCR for mouse *A2M* (*n* = 3).
(DOCX)

S1 Table List of the transcripts modulated by A2M* treatment in the human A549 cell line. TPM counts for regulated transcripts in A2M*-treated cells; explicitly mentioned in the text (*P* < 0.01) and additional (*P* > 0.01). Full list of regulated transcript can be found at GSE 106261.
(DOCX)

S2 Table Correlations of the A549 sample groups against the Cancer RNASeq Nexus. The Pearson correlation coefficients between the average transcript expressions of the A549 A2M*-treated sample groups against the Cancer RNASeq Nexus (CRN), as well as the correlation between the average transcript expressions of the A549 controls (PBS) and the CRN. **(A)** Correlation between the individual stages I through IV of the lung adenocarcinoma samples of the CRN and both sample groups (A2M* and PBS). **(B)** Correlation against the adjacent normal of the CRN against both sample groups.
(DOCX)

S3 Table List of the genes modulated by A2M* treatment in xenograft stroma samples. RPKM counts for regulated genes in tumour stroma samples of A2M*-treated mice; explicitly mentioned in the text, including the top 10 up- and down-regulated genes. Full list of regulated genes can be found at GSE 106989.
(DOCX)

S4 Table List of the genes modulated by A2M* treatment in liver samples. RPKM counts for regulated genes in livers of A2M*-treated mice; explicitly mentioned in the text, including the top 10 up- and down-regulated genes. Full list of regulated genes can be found at GSE 107195.
(DOCX)

S5 Table List of the main pathways modulated by A2M* treatment in liver samples. Observed Pathway Activation Strength (PAS) for regulated pathways in livers of A2M*-treated mice.
(DOCX)

S6 Table List of antibodies. List of primary and secondary antibodies used for Western blotting, immunohistochemistry and flow cytometry.
(DOCX)

S7 Table List of primers. List of forward and reverse primer pairs used for analysis of gene expression by RT-PCR and qPCR.
(DOCX)

S1 Protocol Detailed description of the protocols used for mass spectroscopy, proteomics, impedance spectroscopy and histochemistry. (DOCX)
