# Correction: The anti-tumorigenic activity of A2M—A lesson from the naked mole-rat

**DOI:** 10.1371/journal.pone.0195169

**Published:** 2018-03-26

**Authors:** Susanne Kurz, René Thieme, Ronny Amberg, Marco Groth, Heinz-Georg Jahnke, Philipp Pieroh, Lars-Christian Horn, Marlen Kolb, Klaus Huse, Matthias Platzer, Daniela Volke, Faramarz Dehghani, Anton Buzdin, Kathrin Engel, Andrea Robitzki, Ralf Hoffmann, Ines Gockel, Gerd Birkenmeier

The graphical abstract is omitted from the list of Supporting Information. It can be viewed below.

## Supporting information

S1 File Graphical abstract. (DOCX)
