# Supplementary material for: The anti-tumorigenic activity of A2M—A lesson from the naked mole-rat
Source: PLoS One. 2017 Dec 27;12(12):e0189514. doi: 10.1371/journal.pone.0189514 (PMC5744951; doi:10.1371/journal.pone.0189514)
Supplement: S1 Fig — (a) A2M was purified from human plasma and separated by native PAGE (4–20). (b) Purified native and transformed A2M (A2M*) were separated by Rate-PAGE into native (slow-migrating) and transformed (fast-migrating) A2M. (c) Three purified A2M preparations were separated by SDS-PAGE. The A2M* band was subjected to trypsin digestion and analysed by mass spectrometry to confirm the presence of A2M and to identify further co-migrating proteins. (d) Evidence for receptor binding of A2M*. LRP1 was coated to 96-well plates and incubated with increasing concentrations of A2M and A2M*. Bound A2M/A2M* was detected by HRP-labelled polyclonal anti-A2M-Ig. (e) Receptor-associated protein (RAP) (0–50 nM) inhibits binding of A2M* (10 nM) to immobilized LRP1. (f) Analysis of three A2M preparations for their stimulatory effect on human blood monocytes using the whole blood assay. Heparinized blood was incubated with medium (control), 10 ng/mL LPS and three purified A2M samples (A2M1, A2M2, A2M3), respectively, at 5% CO2, 37°C for 8h. Cells were centrifuged and the supernatant was analysed for TNF-alpha using cytometric bead array (CBA) (n = 3). Alb = albumin; Trf = transferrin, A2M = native A2M, A2M* = transformed A2M, RAP = receptor-associated protein. (DOCX) [file pone.0189514.s001.docx]

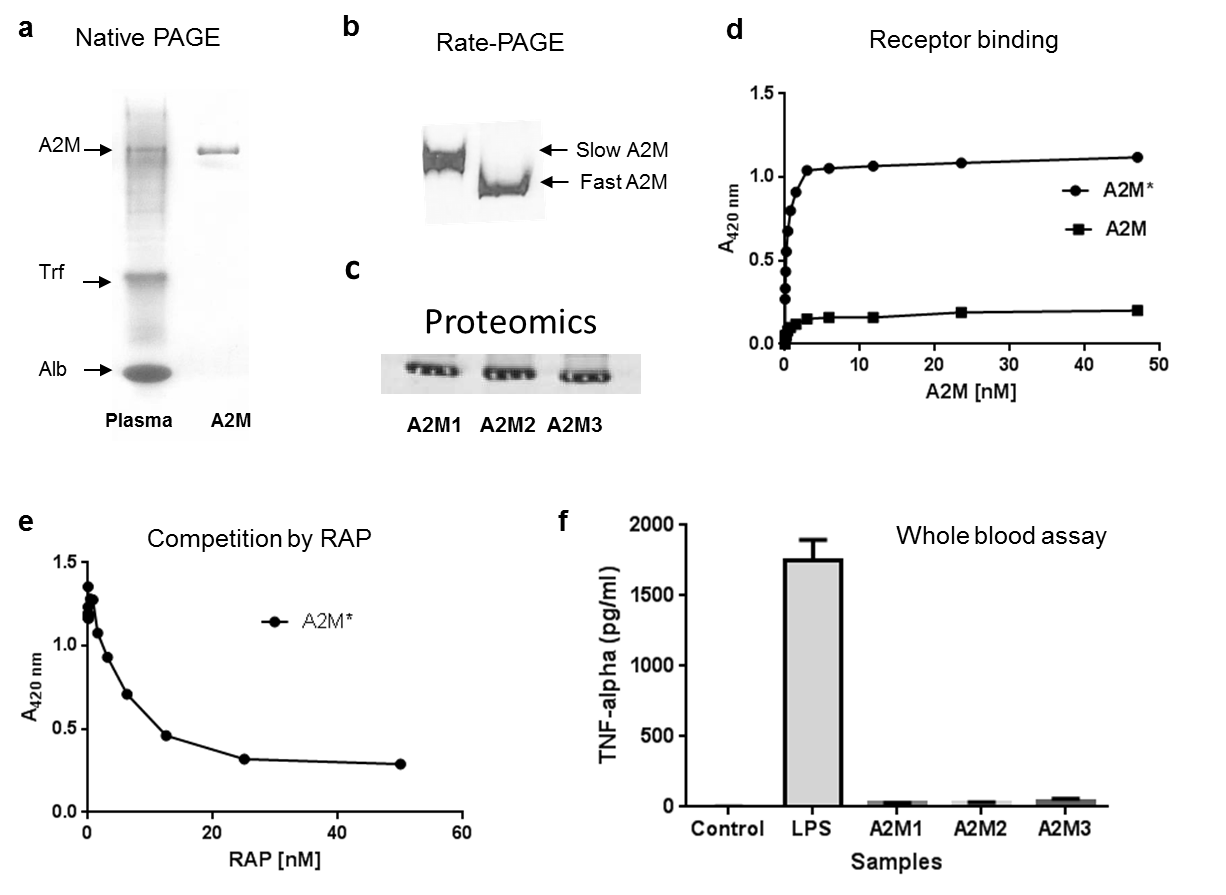


S1 Fig. Purification and quality control of A2M

**(a)** A2M was purified from human plasma and separated by native PAGE (4-20). **(b)** Purified native and transformed A2M (A2M***) were separated by Rate-PAGE into native (slow-migrating) and transformed (fast-migrating) A2M. **(c)** Three purified A2M preparations were separated by SDS-PAGE. The A2M*** band was subjected to trypsin digestion and analysed by mass spectrometry to confirm the presence of A2M and to identify further co-migrating proteins. **(d)** Evidence for receptor binding of A2M*. LRP1 was coated to 96-well plates and incubated with increasing concentrations of A2M and A2M*. Bound A2M/A2M* was detected by HRP-labelled polyclonal anti-A2M-Ig. **(e)** Receptor-associated protein (RAP) (0-50 nM) inhibits binding of A2M* (10 nM) to immobilized LRP1. **(f)** Analysis of three A2M preparations for their stimulatory effect on human blood monocytes using the whole blood assay. Heparinized blood was incubated with medium (control), 10 ng/mL LPS and three purified A2M samples (A2M1, A2M2, A2M3), respectively, at 5% CO_2_, 37°C for 8h. Cells were centrifuged and the supernatant was analysed for TNF-alpha using cytometric bead array (CBA) (*n* = 3). Alb= albumin; Trf = transferrin, A2M = native A2M, A2M* = transformed A2M, RAP = receptor-associated protein.
