# Supplementary material for: The anti-tumorigenic activity of A2M—A lesson from the naked mole-rat
Source: PLoS One. 2017 Dec 27;12(12):e0189514. doi: 10.1371/journal.pone.0189514 (PMC5744951; doi:10.1371/journal.pone.0189514)
Supplement: S2 Fig — (a) Coarse of body weight of tumour-bearing A549 mice treated with A2M* (n = 10) compared to control (n = 9). (b) EDTA blood was withdrawn from A549 tumour bearing mice and analysed in a ScilVet apparatus (ScilVet Animal Care Company, Viernheim, Germany). Blood cells were counted at day 7 after tumour induction (control) and day 31 after A2M* treatment. WBC–white blood cells, RBC–red blood cells, HGB—Hemoglobin, HCT–Hematocrit value, MCV–mean corpuscular volume, MCH–mean corpuscular hematocrit, PLT—platelets, MPV–mean platelet volume, RDW–red cell distribution width, LYM–Lymphocytes, MO—Monocytes, GRA—Granulocytes, (n = 9), (* P < 0.05, **P < 0.01, ***P < 0.001). (c), Effect of A2M* on mouse spleen cells. Spleen cells from A549 tumour-bearing mice treated with A2M* were isolated, stimulated with 10 nM lipopolysaccharide (LPS) or PBS (control) and cytokines were measured by cytokine bead arrays (CBA). (n = 10) (**P < 0.01). Error bars represent mean ± s.d. (DOCX) [file pone.0189514.s002.docx]

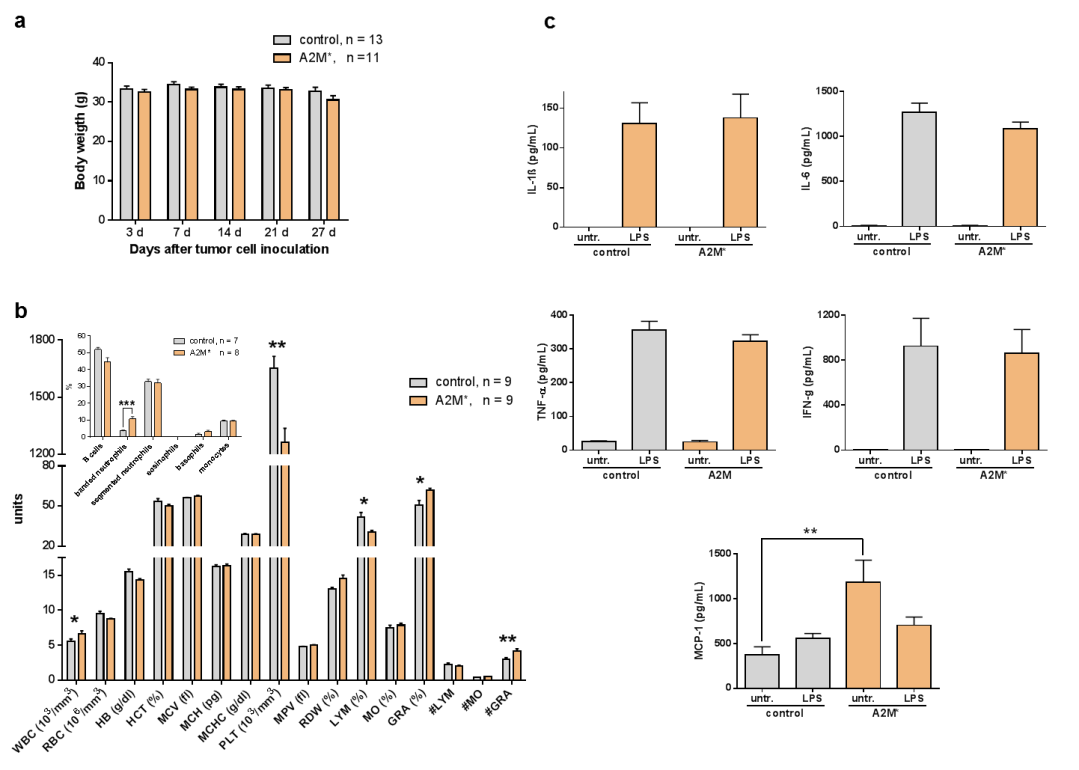


S2 Fig. Analysis of blood cells in tumour-bearing mice before and after treatment with A2M

**(a)** Coarse of body weight of tumour-bearing A549 mice treated with A2M* (*n* = 10) compared to control (*n* = 9). **(b)** EDTA blood was withdrawn from A549 tumour bearing mice and analysed in a ScilVet apparatus (ScilVet Animal Care Company, Viernheim, Germany). Blood cells were counted at day 7 after tumour induction (control) and day 31 after A2M* treatment. WBC – white blood cells, RBC – red blood cells, HGB - Hemoglobin, HCT – Hematocrit value, MCV – mean corpuscular volume, MCH – mean corpuscular hematocrit, PLT - platelets, MPV – mean platelet volume, RDW – red cell distribution width, LYM – Lymphocytes, MO - Monocytes, GRA - Granulocytes, (*n* = 9), (* *P < 0.05*, ***P < 0.01*, ****P < 0.001*). **(c),** Effect of A2M* on mouse spleen cells. Spleen cells from A549 tumour-bearing mice treated with A2M* were isolated, stimulated with 10 nM lipopolysaccharide (LPS) or PBS (control) and cytokines were measured by cytokine bead arrays (CBA). (*n* = 10) (***P < 0.01*). Error bars represent mean ± s.d.
