# Supplementary material for: The anti-tumorigenic activity of A2M—A lesson from the naked mole-rat
Source: PLoS One. 2017 Dec 27;12(12):e0189514. doi: 10.1371/journal.pone.0189514 (PMC5744951; doi:10.1371/journal.pone.0189514)
Supplement: S4 Fig — Hematoxilin-eosin (HE) stained A549 tumour slices obtained from PBS-treated animals (control, a-d) and A2M*-treated animals (e-h). (a) Peripheral compartment of PBS treated tumour in overview. (b) Compact tumour organization with a few cells yielding apoptotic signs. (c) Tumour cells in a small area of tumour destruction (+) and cells with signs of apoptosis (arrow). (d) Dispersed vital A549 cells with few cells showing signs of degradation. (e) Peripheral compartment of an A2M*-treated tumour in overview. (f) Necrotic area (*) with macrophage accumulation the tumour tissue (arrow). (g) Low number of vital tumour cells paralleled by massive loss of tumour cytoarchitecture. (h) Loss of tumour tissue (*) accompanied by accumulation of macrophages (arrow). Scale bar: 300 μm (a and e), 100 μm (b-d, f-h). (DOCX) [file pone.0189514.s004.docx]

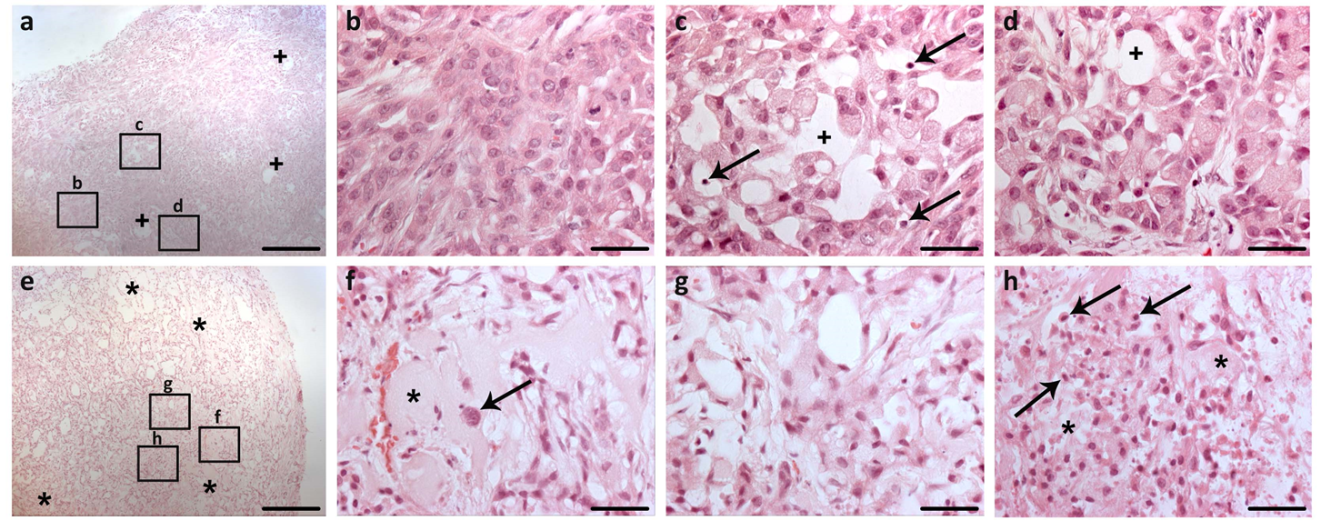


S4 Fig. Morphological analysis of tumour tissue

Hematoxilin-eosin (HE) stained A549 tumour slices obtained from PBS-treated animals (control, a-d) and A2M*-treated animals (e-h). **(a)** Peripheral compartment of PBS treated tumour in overview. **(b)** Compact tumour organization with a few cells yielding apoptotic signs. **(c)** Tumour cells in a small area of tumour destruction (+) and cells with signs of apoptosis (arrow). **(d)** Dispersed vital A549 cells with few cells showing signs of degradation. **(e)** Peripheral compartment of an A2M*-treated tumour in overview. **(f)** Necrotic area (*) with macrophage accumulation the tumour tissue (arrow). **(g)** Low number of vital tumour cells paralleled by massive loss of tumour cytoarchitecture. **(h)** Loss of tumour tissue (*) accompanied by accumulation of macrophages (arrow). Scale bar: 300 µm **(a and e)**, 100 µm **(b-d, f-h)**
