# Supplementary material for: The anti-tumorigenic activity of A2M—A lesson from the naked mole-rat
Source: PLoS One. 2017 Dec 27;12(12):e0189514. doi: 10.1371/journal.pone.0189514 (PMC5744951; doi:10.1371/journal.pone.0189514)
Supplement: S5 Fig — (a-c) Liver of scarified mice were homogenized and analysed for A2M protein content and RNA by qRT-PCR and Western blotting. (d) Balb/c mice were injected with A2M* (5.6 mg/20g body weight), sacrificed after indicated times and the expression of mice A2M in the liver was analysed by qRT-PCR (n = 3 for each time point). (e) Balb/c mice were given a bolus injection of zinc orotate (0.5 mg/kg) (SigmaAldrich), and mouse A2M gene expression in the liver was determined by qRT-PCR. (f) Primary murine hepatocyte cultures from Balb/c mice were stimulated with native and transformed human A2M* (0–100 nM) for 24h followed by qRT-PCR for mouse A2M (n = 3). (DOCX) [file pone.0189514.s005.docx]

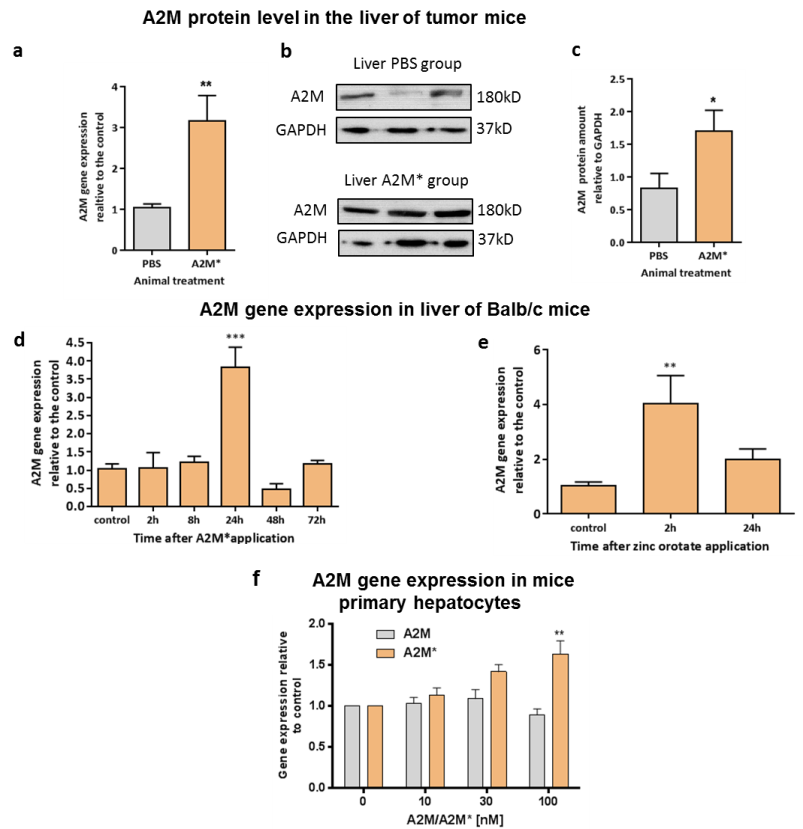


S5 Fig. Effect of A2M* on expression of endogenous mouse A2M in the liver of A549-xenografted mice, Balb/c mice and isolated hepatocytes

**(a-c)** Liver of scarified mice were homogenized and analysed for A2M protein content and RNA by qRT-PCR and Western blotting. **(d)** Balb/c mice were injected with A2M* (5.6 mg/20g body weight), sacrificed after indicated times and the expression of mice A2M in the liver was analysed by qRT-PCR (*n* = 3 for each time point). **(e)** Balb/c mice were given a bolus injection of zinc orotate (0.5 mg/kg) (SigmaAldrich), and mouse *A2M* gene expression in the liver was determined by qRT-PCR**. (f)** Primary murine hepatocyte cultures from Balb/c mice were stimulated with native and transformed human A2M* (0-100 nM) for 24h followed by qRT-PCR for mouse *A2M* (*n* =3).
