# Supplementary material for: The anti-tumorigenic activity of A2M—A lesson from the naked mole-rat
Source: PLoS One. 2017 Dec 27;12(12):e0189514. doi: 10.1371/journal.pone.0189514 (PMC5744951; doi:10.1371/journal.pone.0189514)
Supplement: S1 Table — TPM counts for regulated transcripts in A2M*-treated cells; explicitly mentioned in the text (P < 0.01) and additional (P > 0.01). Full list of regulated transcript can be found at GSE 106261. (DOCX) [file pone.0189514.s006.docx]

S1 Table. List of the transcripts modulated by A2M* treatment in the human A549 cell line.

TPM counts for regulated transcripts in A2M*-treated cells; explicitly mentioned in the text (*P* < 0.01) and additional (*P* > 0.01). Full list of regulated transcript can be found at GSE 106261.

| Gene with regulated Transcript | Longevity | Tumor Supressor | Tumor Progrssor | WNT | Mitosis / Spindle Cell Cycle | DNA Damage | Biomarker | Cell Proliferation / Apoptosis | Untreated | Treated | log_2_ FC | pValue |
| --- | --- | --- | --- | --- | --- | --- | --- | --- | --- | --- | --- | --- |
| CYP2U1 |  |  | x |  |  |  |  |  | 1.000 ± 0.769 | 0.000 ± 0.000 | - | 0.02250 |
| CBY1 |  |  |  | x |  |  |  |  | 0.821 ± 0.771 | 0.043 ± 0.071 | -4.27072 | 0.04939 |
| ATP7A |  |  |  |  |  |  |  | x | 2.263 ± 1.741 | 0.203 ± 0.521 | -3.48079 | 0.03530 |
| LRP12 |  |  |  |  |  |  |  |  | 4.405 ± 2.146 | 0.594 ± 0.696 | -2.89034 | 0.00964 |
| miR-1296 |  |  |  |  |  |  |  |  | 1.017 ±0.262 | 0.168 ±0.279 | -2.60014 | 0.00036 |
| PREP | x |  |  |  |  |  |  |  | 6.823 ± 2.064 | 1.350 ± 2.092 | -2.33704 | 0.00110 |
| LIN9 |  |  |  |  | x |  |  | x | 5.782 ± 2.399 | 1.149 ± 1.44 | -2.33098 | 0.00280 |
| NDE1 |  |  |  |  | x |  |  |  | 0.796 ± 0.385 | 0.181 ± 0.296 | -2.13658 | 0.00916 |
| ANXA10 |  |  |  |  | x |  |  |  | 2.251 ± 0.991 | 0.570 ± 0.434 | -1.98110 | 0.00804 |
| NT5E (CD73) |  |  |  |  |  |  |  | x | 3.929 ± 1.394 | 1.090 ± 0.764 | -1.84977 | 0.00281 |
| ACSM3 |  |  |  |  |  |  |  |  | 1.025 ±0.251 | 0.287 ±0.240 | -1.83515 | 0.00046 |
| YWHAZ |  |  |  | x |  |  |  |  | 7.413 ± 5.426 | 2.117 ± 1.544 | -1.80787 | 0.06066 |
| HDGF |  |  |  |  |  |  |  | x | 6.178 ± 3.710 | 2.245 ± 2.537 | -1.46033 | 0.04980 |
| RN7SL620P |  |  |  |  |  |  |  |  | 1.041 ±0.303 | 0.432 ±0.313 | -1.27060 | 0.00581 |
| RANGRF (MOG1) |  |  |  |  |  |  |  |  | 1.409 ±0.388 | 0.589 ±0.172 | -1.25841 | 0.00321 |
| miR-548v |  |  |  |  |  |  |  |  | 3.045 ± 0.999 | 1.362 ± 0.449 | -1.16088 | 0.00844 |
| BOP1 |  |  | x |  | x |  |  |  | 9.321 ± 4.427 | 4.182 ± 3.222 | -1.15645 | 0.03503 |
| ZPR1 |  |  |  |  |  |  |  | x | 5.819 ± 1.040 | 2.635 ± 1.77 | -1.14279 | 0.00480 |
| NUP88 |  |  |  |  | x |  |  |  | 21.070 ± 8.890 | 11.162 ± 3.619 | -0.91664 | 0.03655 |
| MASTL |  |  |  |  | x |  |  | x | 9.983 ± 2.192 | 6.566 ± 3.158 | -0.60456 | 0.04737 |
| HBS1L |  | x |  |  |  |  |  | x | 1.262 ± 0.841 | 2.200 ± 0.204 | 0.80205 | 0.04006 |
| RBL2 |  |  |  |  | x |  |  |  | 5.238 ± 5.786 | 11.177 ± 2.467 | 1.09342 | 0.04893 |
| THTPA |  |  |  |  |  |  |  | x | 0.723 ± 0.512 | 1.577 ± 0.412 | 1.12597 | 0.00813 |
| CDK2AP1 |  |  |  |  | x |  |  |  | 25.08 ± 23.55 | 56.817 ± 10.13 | 1.17943 | 0.01943 |
| CDK5RAP3 |  |  |  | x |  |  |  |  | 2.412 ± 2.367 | 6.006 ± 2.884 | 1.31617 | 0.03415 |
| FAM57A (CT120) |  |  |  |  | x |  |  |  | 0.783 ± 0.705 | 2.931 ± 1.240 | 1.90479 | 0.00560 |
| RMI1 |  | x |  |  | x |  |  |  | 1.925 ± 1.554 | 7.399 ± 2.437 | 1.94213 | 0.00162 |
| ARHGAP29 (PARG1) |  |  | x |  |  |  |  |  | 0.959 ± 1.241 | 4.406 ± 1.782 | 2.20022 | 0.00309 |
| RSF1 |  |  |  |  |  |  |  |  | 0.279 ±0.309 | 1.328 ±0.635 | 2.24888 | 0.00828 |
| ZSCAN22 |  |  |  |  |  |  |  |  | 0.195 ±0.383 | 1.016 ±0.320 | 2.38321 | 0.00224 |
| WIPI2 (ATG18) |  |  |  |  |  |  |  |  | 0.308 ±0.578 | 1.691 ±0.621 | 2.45801 | 0.00248 |
| EIF2B3 | x |  |  |  |  |  |  |  | 0.371 ± 0.27 | 2.05 ± 0.644 | 2.46588 | 0.00078 |
| DAPK1 |  |  |  |  |  |  |  | x | 0.200 ± 0.397 | 1.253 ± 0.662 | 2.64937 | 0.00900 |
| ZNF786 |  |  |  |  |  |  |  |  | 0.211 ±0.517 | 2.101 ±1.218 | 3.31433 | 0.00996 |
| UBAP2L |  |  |  |  |  |  |  |  | 0.124 ±0.343 | 1.745 ±0.887 | 3.81947 | 0.00445 |
| LDLRAD3 |  | x |  |  |  |  |  |  | 0.000 ± 0.000 | 2.098 ± 1.910 | - | 0.03700 |
