# Supplementary material for: The anti-tumorigenic activity of A2M—A lesson from the naked mole-rat
Source: PLoS One. 2017 Dec 27;12(12):e0189514. doi: 10.1371/journal.pone.0189514 (PMC5744951; doi:10.1371/journal.pone.0189514)
Supplement: S2 Table — The Pearson correlation coefficients between the average transcript expressions of the A549 A2M*-treated sample groups against the Cancer RNASeq Nexus (CRN), as well as the correlation between the average transcript expressions of the A549 controls (PBS) and the CRN. (A) Correlation between the individual stages I through IV of the lung adenocarcinoma samples of the CRN and both sample groups (A2M* and PBS). (B) Correlation against the adjacent normal of the CRN against both sample groups. (DOCX) [file pone.0189514.s007.docx]

S2 Table. Correlations of the A549 sample groups against the Cancer RNASeq Nexus.

The Pearson correlation coefficients between the average transcript expressions of the A549 A2M*-treated sample groups against the Cancer RNASeq Nexus (CRN), as well as the correlation between the average transcript expressions of the A549 controls (PBS) and the CRN. **(A)** Correlation between the individual stages I through IV of the lung adenocarcinoma samples of the CRN and both sample groups (A2M* and PBS). **(B)** Correlation against the adjacent normal of the CRN against both sample groups.

| **A** | | | | | | |
| --- | --- | --- | --- | --- | --- | --- |
| Cancer Stages | | Correlation | |  | absolute change | relative change |
| Stages | #Samples in CRN | PBS | A2M* |  | \|A2M\|-\|PBS\| | 1-PBS/A2M* |
| I | 5 | -0.04874 | -0.04514 |  | -0.00360 | -0.07979 |
| IA | 133 | 0.60594 | 0.60987 |  | 0.00393 | 0.00645 |
| IB | 140 | 0.65970 | 0.66419 |  | 0.00449 | 0.00676 |
| IIA | 51 | 0.64248 | 0.64144 |  | -0.00104 | -0.00163 |
| IIB | 73 | 0.78623 | 0.78690 |  | 0.00067 | 0.00086 |
| IIIA | 73 | 0.75686 | 0.75654 |  | -0.00033 | -0.00043 |
| IIIB | 11 | 0.45101 | 0.43425 |  | -0.01676 | -0.03859 |
| IV | 27 | 0.40428 | 0.40143 |  | -0.00285 | -0.00710 |
| Averages: |  | 0.53222 | 0.53118 |  | -0.2% | -1.4% |
| **B** | | | | | | |
| Adjacent Normal | | Correlation | |  | absolute change | relative change |
| Stage | #Samples in CRN | PBS | A2M* |  | \|A2M\|-\|PBS\| | 1-PBS/A2M* |
| I | 59 | 0.04355 | 0.06161 |  | 0.01806 | 0.29312 |
| IA | 59 | 0.46029 | 0.48129 |  | 0.02101 | 0.04364 |
| IB | 59 | 0.44888 | 0.46597 |  | 0.01709 | 0.03667 |
| IIA | 59 | 0.45480 | 0.48692 |  | 0.03212 | 0.06597 |
| IIB | 59 | 0.61451 | 0.61974 |  | 0.00523 | 0.00844 |
| IIIA | 59 | 0.59341 | 0.61178 |  | 0.01837 | 0.03002 |
| IIIB | 59 | 0.14543 | 0.19978 |  | 0.05434 | 0.27202 |
| IV | 59 | 0.30015 | 0.31108 |  | 0.01094 | 0.03515 |
| Averages: |  | 0.38263 | 0.40477 |  | 2.2% | 9.8% |
