# Supplementary material for: The anti-tumorigenic activity of A2M—A lesson from the naked mole-rat
Source: PLoS One. 2017 Dec 27;12(12):e0189514. doi: 10.1371/journal.pone.0189514 (PMC5744951; doi:10.1371/journal.pone.0189514)
Supplement: S3 Table — RPKM counts for regulated genes in tumour stroma samples of A2M*-treated mice; explicitly mentioned in the text, including the top 10 up- and down-regulated genes. Full list of regulated genes can be found at GSE 106989. (DOCX) [file pone.0189514.s008.docx]

S3 Table. List of the genes modulated by A2M* treatment in xenograft stroma samples.

RPKM counts for regulated genes in tumour stroma samples of A2M*-treated mice; explicitly mentioned in the text, including the top 10 up- and down-regulated genes. Full list of regulated genes can be found at GSE106989.

| Regulated Gene | Untreated | Treated | log_2_ FC | pValue (DESeq) | pValue (edgeR) |
| --- | --- | --- | --- | --- | --- |
| CRABP1 | 17.146 ± 10.672 | 2.877 ± 2.857 | -2.6671 | 1.25E-06 | 3.25E-20 |
| ELOVL3 | 5.219 ± 3.956 | 0.93 ± 0.54 | -2.5323 | 6.70E-06 | 1.75E-16 |
| PTPRZ1 | 0.944 ± 0.628 | 0.231 ± 0.189 | -2.1122 | 8.84E-05 | 2.37E-11 |
| PRG4 | 23.526 ± 12.505 | 6.017 ± 7.141 | -1.9969 | 7.34E-05 | 5.73E-19 |
| LUM | 119.274 ± 36.623 | 31.476 ± 14.959 | -1.9533 | 3.38E-17 | 3.92E-19 |
| MIAT | 1.42 ± 0.715 | 0.384 ± 0.171 | -1.9307 | 4.13E-07 | 6.53E-12 |
| EFEMP1 | 43.537 ± 9.54 | 13.422 ± 6.809 | -1.7445 | 5.79E-13 | 1.11E-14 |
| SFRP4 | 36.08 ± 19.547 | 11.176 ± 6.386 | -1.7283 | 4.71E-06 | 3.17E-14 |
| DIO2 | 12.447 ± 5.429 | 4.156 ± 2.309 | -1.6368 | 9.22E-07 | 9.17E-13 |
| FIGF | 7.138 ± 1.926 | 2.378 ± 1.737 | -1.6231 | 2.74E-06 | 1.63E-09 |
| INPP5J | 0.127 ± 0.101 | 0.917 ± 0.268 | 2.2187 | 7.73E-05 | 9.07E-10 |
| GFI1 | 0.42 ± 0.217 | 2.13 ± 0.841 | 2.2829 | 3.89E-08 | 5.15E-12 |
| PRF1 | 1.305 ± 0.976 | 6.652 ± 2.805 | 2.2861 | 4.48E-06 | 9.05E-16 |
| SH2D2A | 1.648 ± 0.777 | 8.376 ± 1.51 | 2.3023 | 2.41E-25 | 6.15E-21 |
| DSCAM | 0.092 ± 0.054 | 0.472 ± 0.085 | 2.3118 | 3.22E-05 | 9.01E-09 |
| IFNG | 1.505 ± 1.516 | 7.913 ± 3.601 | 2.3349 | 2.46E-06 | 1.55E-14 |
| TIGIT | 1.774 ± 1.229 | 9.644 ± 4.457 | 2.3881 | 4.12E-06 | 1.64E-14 |
| TOX | 0.772 ± 0.384 | 4.395 ± 1.631 | 2.4487 | 3.52E-09 | 5.89E-17 |
| ICOS | 0.456 ± 0.755 | 2.701 ± 1.565 | 2.5518 | 1.37E-05 | 7.47E-16 |
| ILDR1 | 0.282 ± 0.337 | 1.343 ± 0.718 | 2.7943 | 2.64E-05 | 2.68E-09 |
