# Supplementary material for: The anti-tumorigenic activity of A2M—A lesson from the naked mole-rat
Source: PLoS One. 2017 Dec 27;12(12):e0189514. doi: 10.1371/journal.pone.0189514 (PMC5744951; doi:10.1371/journal.pone.0189514)
Supplement: S4 Table — RPKM counts for regulated genes in livers of A2M*-treated mice; explicitly mentioned in the text, including the top 10 up- and down-regulated genes. Full list of regulated genes can be found at GSE 107195. (DOCX) [file pone.0189514.s009.docx]

S4 Table. List of the genes modulated by A2M* treatment in liver samples.

RPKM counts for regulated genes in livers of A2M*-treated mice; explicitly mentioned in the text, including the top 10 up- and down-regulated genes. Full list of regulated genes can be found at GSE 107195.

| Regulated Gene | Untreated | Treated | log_2_ FC | pValue (DESeq) | pValue (edgeR) |
| --- | --- | --- | --- | --- | --- |
| CDKN1A | 9.649 ± 3.494 | 0.478 ± 0.233 | -4.3599 | 7.96E-21 | 9.83E-54 |
| ARNTL | 6.741 ± 5.304 | 0.414 ± 0.256 | -4.0149 | 6.78E-08 | 1.63E-48 |
| MTHFR | 4.223 ± 2.932 | 0.475 ± 0.184 | -3.1503 | 6.46E-08 | 7.98E-35 |
| NCKAP5 | 0.53 ± 0.234 | 0.081 ± 0.068 | -2.6914 | 7.94E-14 | 7.71E-21 |
| COL5A3 | 2.793 ± 1.882 | 0.63 ± 0.325 | -2.1462 | 7.10E-06 | 2.80E-18 |
| TUBB2B | 22.678 ± 9.377 | 5.494 ± 1.868 | -2.0571 | 1.04E-09 | 1.01E-17 |
| TUBB2A | 74.492 ± 30.487 | 18.106 ± 5.809 | -2.0510 | 3.07E-10 | 6.43E-18 |
| PDK4 | 2.161 ± 0.855 | 0.543 ± 0.394 | -1.9997 | 4.97E-09 | 8.30E-15 |
| CHKA | 19.333 ± 11.417 | 5.296 ± 1.284 | -1.8655 | 2.97E-06 | 2.93E-15 |
| PNRC1 | 40.76 ± 20.446 | 12.26 ± 4.539 | -1.7399 | 4.16E-07 | 1.55E-13 |
| CYP4A31 | 0.993 ± 0.62 | 2.669 ± 1.202 | 1.4215 | 5.04E-06 | 8.28E-09 |
| SLC5A6 | 2.194 ± 0.625 | 6.173 ± 1.755 | 1.4800 | 6.97E-13 | 2.47E-10 |
| PER2 | 2.017 ± 1.595 | 5.817 ± 2.444 | 1.5067 | 5.82E-05 | 7.79E-11 |
| POR | 47.119 ± 13.731 | 137.173 ± 40.771 | 1.5139 | 2.64E-12 | 3.12E-11 |
| TEF | 11.051 ± 10.227 | 40.16 ± 8.831 | 1.8261 | 2.97E-13 | 2.17E-15 |
| THRSP | 170.104 ± 198.598 | 667.082 ± 246.003 | 1.9285 | 9.22E-07 | 5.03E-17 |
| PER3 | 0.667 ± 0.772 | 3.703 ± 0.699 | 2.4357 | 5.13E-21 | 1.30E-23 |
| CIART | 1.849 ± 2.841 | 11.124 ± 5.038 | 2.5451 | 2.67E-09 | 8.26E-25 |
| DBP | 3.443 ± 5.924 | 33.343 ± 10.837 | 3.2339 | 3.90E-19 | 1.04E-38 |
| WEE1 | 0.432 ± 0.302 | 4.203 ± 2.395 | 3.2767 | 7.12E-10 | 1.48E-35 |
| USP2 | 0.942 ± 1.09 | 10.595 ± 3.446 | 3.4556 | 1.11E-26 | 1.57E-42 |
