# Supplementary material for: The anti-tumorigenic activity of A2M—A lesson from the naked mole-rat
Source: PLoS One. 2017 Dec 27;12(12):e0189514. doi: 10.1371/journal.pone.0189514 (PMC5744951; doi:10.1371/journal.pone.0189514)
Supplement: S5 Table — Observed Pathway Activation Strength (PAS) for regulated pathways in livers of A2M*-treated mice. (DOCX) [file pone.0189514.s010.docx]

S5 Table. List of the main pathways modulated by A2M* treatment in liver samples.

Observed Pathway Activation Strength (PAS) for regulated pathways in livers of A2M*-treated mice.

| Regulated Main Pathway | Mean PAS | Standard Error PAS | pValue |
| --- | --- | --- | --- |
| MAPK Signaling Main Pathway | -7.52068 | 0.80626 | 0.01052 |
| Circadian Main Pathway | -6.86191 | 0.76102 | 0.01052 |
| ILK Signaling Main Pathway | -6.59052 | 1.21397 | 0.01052 |
| ERK Signaling Main Pathway | -5.87641 | 0.89089 | 0.01052 |
| GPCR Main Pathway | -5.38263 | 0.38343 | 0.01052 |
| JNK Main Pathway | -5.20554 | 0.81585 | 0.01052 |
| JAK-STAT Main Pathway | -5.01587 | 0.53904 | 0.01052 |
| Ras Main Pathway | -4.14873 | 0.47569 | 0.01052 |
| cAMP Main Pathway | -4.09454 | 0.57949 | 0.01052 |
| CREB Main Pathway | -3.72129 | 0.6349 | 0.01052 |
| TGF-Beta Main Pathway | -3.69485 | 0.49136 | 0.01052 |
| BRCA1 Main Pathway | -3.48843 | 0.47474 | 0.01052 |
| PTEN Main Pathway | -3.23955 | 0.7439 | 0.03301 |
| IGF1R Signaling Main Pathway | -3.01841 | 0.31506 | 0.01052 |
| IL-2 Main Pathway | -2.95604 | 0.43647 | 0.01052 |
| PAK Main Pathway | -2.71099 | 0.70829 | 0.01052 |
| IP3 Main Pathway | -2.62450 | 0.45792 | 0.01902 |
| Estrogen Main Pathway | -2.53794 | 0.67881 | 0.01052 |
| WNT Main Pathway | -2.43842 | 0.32898 | 0.01052 |
| Erythropoietin Main Pathway | -2.24162 | 0.37229 | 0.01052 |
| ATM Main Pathway | -1.78683 | 0.18283 | 0.01052 |
| PPAR Main Pathway | -1.50129 | 0.28639 | 0.01902 |
| HGF Main Pathway | -1.48848 | 0.16014 | 0.01052 |
| Chromatin Main Pathway | -1.07026 | 0.11312 | 0.01052 |
| p53 Signaling Main Pathway | 0.85822 | 0.25745 | 0.01052 |
| Caspase Cascade Main Pathway | 2.17206 | 0.57434 | 0.01902 |
| Cellular Apoptosis Main Pathway | 3.68850 | 0.67651 | 0.01052 |
