# Supplementary material for: The anti-tumorigenic activity of A2M—A lesson from the naked mole-rat
Source: PLoS One. 2017 Dec 27;12(12):e0189514. doi: 10.1371/journal.pone.0189514 (PMC5744951; doi:10.1371/journal.pone.0189514)
Supplement: S6 Table — List of primary and secondary antibodies used for Western blotting, immunohistochemistry and flow cytometry. (DOCX) [file pone.0189514.s011.docx]

**S6 Table. List of antibodies**

| **Primary antibodies** | | | | | | |
| --- | --- | --- | --- | --- | --- | --- |
| **Antigen** | **Species** | **Mono/Poly** | **Cat.No.** | **Source** | **Dilution** | **Label** |
|  |  |  |  |  |  |  |
| hAKT | rabbit | mono | 4691 | Cell Signaling | 1:2000 |  |
| hP-AKT(Ser473) | rabbit | mono | 4060 | Cell Signaling | 1:1000 |  |
| hP-AKT(Thr133) | rabbit | mono | 244F9 | Cell Signaling | 1.1000 |  |
| hCD44 | mouse | mono | 3570 | Cell Signaling | 1:1000 |  |
| hE-Cadherin | mono | mouse | 5296 | Cell Signaling | 1:1000 |  |
| hCD29 | rabbit | poly | 4706 | Cell Signaling | 1:1000 |  |
| hGSK3-ß | rabbit | mono | 9315 | Cell Signaling | 1:1000 |  |
| hP-GSK3ß (Ser9) | rabbit | Mono | 9322 | Cell Signaling | 1:1000 |  |
| hBad | rabbit | Mono | 9239 | Cell Signaling | 1:1000 |  |
| hP-Bad(Ser136) | rabbit | Mono | 4366 | Cell Signaling | 1:1000 |  |
| hPTEN | rabbit | mono | 9559S | Cell Signaling | 1:1000 |  |
| hSNAIL | rabbit | mono | 3879S | Cell SIgnaling | 1:1000 |  |
| hSmad2/3 | rabbit | mono | 5678 | Cell Signaling | 1:1000 |  |
| hGAPDH | rabbit | mono | 5174 | Cell Signaling | 1:1000 |  |
| hP-Smad2 (Ser465/467)/3(Ser423/425) | rabbit | mono | 8828 | Cell Signaling | 1:1000 |  |
| hCleaved Caspase 3 | rabbit | poly | 9661 | Cell Signaling | 1:200 |  |
| hIba1 | rabbit | poly | 19741 | Wako | 1:200 |  |
| hß-Catenin | mouse | mono | 610153 | BD Bioscience | 1:1000 |  |
| hVimentin | mouse | mono | V6389 | SigmaAldrich | 1:5000 |  |
| hEpCAM | mouse | mono | Sc-25308 | Santa Cruz | 1:500 |  |
| hKi67i | rabbit | mono | Ki681CO1 | DCS Diagnostics | 1:200 |  |
| hß-Actin | mouse | mono | AM1829b | Abgent | 1:2000 |  |
| hFOXO3a | mouse | mono | AO2173a) | Abgent | 1:1000 |  |
| hP-FOXO3 (SER 418) | rabbit | poly | AP3731a | Abgent | 1:500 |  |
| hLRP1, a-chain | mouse | mono | #02-03 | Biomac | 1:1000 |  |
| hLRP1, ß-chain | mouse | mono | #02-04 | Biomac | 1:1000 |  |
| hRAP | mouse | mono | #02-05 | Biomac | 1:1000 |  |
| hA2M | rabbit | poly | #01-01 | Biomac | 1:5000 |  |
| hA2M native | mouse | mono | #02-01 | Biomac | 1:1000 |  |
| hA2M* | mouse | mono | #02-02 | Biomac | 1:1000 |  |
| hA2M | rabbit | poly | #03-01 | Biomac | 1:2000 | HRP |
| hGlo1 | mouse | mono | #02-14 | Biomac | 1:2000 |  |
| CD44 | mouse | mono | 550989 | BD Bioscience | 1:50 | PE |
| Isotype IgG1 | mouse | mono | 555749 | BD Bioscience | 1:50 | PE |
| **Secondary antibodies** | | | | | | |
| Rabbit-Ig | goat | poly | 111135146 | Dianova | 1:7500 | HRP |
| Mouse-Ig | goat | poly | P0447 | DAKO | 1:5000 | HRP |
| Rabbit-Ig | goat | poly | E043201 | DAKO | 1:800 | Biotin |
| Mouse-Ig | rabbit | poly | Ab97044 | Abcam | 1:2000 | Biotin |
