# Supplementary material for: The anti-tumorigenic activity of A2M—A lesson from the naked mole-rat
Source: PLoS One. 2017 Dec 27;12(12):e0189514. doi: 10.1371/journal.pone.0189514 (PMC5744951; doi:10.1371/journal.pone.0189514)
Supplement: S7 Table — List of forward and reverse primer pairs used for analysis of gene expression by RT-PCR and qPCR. (DOCX) [file pone.0189514.s012.docx]

**S7 Table. List of Primers**

| **RT-PCR Primer** | | | |
| --- | --- | --- | --- |
| **Gene** | **Forward** | **Reverse** | **Amplicon** |
| hCD44(forms) | TCCCAGACGAAGACAGTCCCTGGAT | CACTGGGGTGGAATGTGTCTTGGTC | 479,83bp |
| hSNAI1 | ACAGTGGGAAAGGCTCCCAGC | GGTTGGAGCGGTCAGCGAAGG | 384bp |
| hGAPDH | AAGGGTCATCATCTCTGCCC | ATGATGTTCTGGAGAGCCCC | 270bp |
|  |  |  |  |
| **qPCR Primer** | | | |
| **Gene** | **Forward** | **Reverse** |  |
| hE-Cadherin | CAGCACGTACACAGCCCTAA | GGCGTTGTCATTCACATCAG | 103bp |
| hCD44 | GCAGTCAACAGTCGAAGAAGG | TGTCCTCCACAGCTCCATT | 76bp |
| hVimentin | ACTTTTCCTCCCTGAACCTGAG | AAGGTCATCGTGATGCTGAGAA | 146bp |
| hGAPDH | TCAACGGATTTGGTCGTATTGG | GCAACAATATCCACTTTACCAGA | 73bp |
| hbeta-Actin | GTCTTCCCCTCCATCGTG | AGGGTGAGGATGCCTCTCTT | 113bp |
| mA2M | CACAAATGCCTCAGCACCAC | CTTTGGTTCCCACGGACAGA | 156bp |
| mbeta-Actin | GTCTTCCCCTCCATCGTG | AGGGTGAGGATGCCTCTCTT | 113bp |
|  |  |  |  |
| **miR21 analysis Primer** | | | |
| **Gene** | **Forward** | **Reverse** |  |
| Stem loop | GTCGTATCCAGTGCAGGGTCCGAGGTATTCGCACTGGATACGACTCAACA | |  |
| hU6 | CTCGCTTCGGCAGCACA | AACGCTTCACGAATTTGCGT | 94bp |
| hpri-miR21 | TTTTGTTTTGCTTGGGAGGA | AGCAGACAGTCAGGCAGGAT |  |
| hmiR-21 | GCCCGCTAGCTTATCAGACTGATG | GTGCAGGGTCCGAGGT | 62bp |
