# Supplementary material for: The anti-tumorigenic activity of A2M—A lesson from the naked mole-rat
Source: PLoS One. 2017 Dec 27;12(12):e0189514. doi: 10.1371/journal.pone.0189514 (PMC5744951; doi:10.1371/journal.pone.0189514)
Supplement: S1 Protocol — (DOCX) [file pone.0189514.s013.docx]

**S1 Protocol.**

**Mass spectrometry**

Gel material from the *A2M* bands was collected with an ExQuest Spot Cutter (Bio-Rad GmbH, Munich). Gel plugs were de-stained, digested with trypsin (SERVA Electrophoresis GmbH, 37°C, 18h), and analyzed by nanoRP-HPLC-QqTOF-MS/MS. Briefly, tryptic peptides were dissolved in aqueous acetonitrile (3%) containing formic acid (0.1 %) and analyzed on a Waters ACQUITY Ultra Performance LC™ system (Symmetry® C18-trap and nanoACQUITY® BEH C18-column 75 µm x 100mm) coupled on-line to a Q-TOF Synapt GS2i MS (Waters, MS Technologies, Manchester, UK). Column temperature was set to 35 °C. Peptides were eluted at a flow rate of 0.3 µL/min using a linear gradient from 3 % to 40 % aqueous acetonitrile (0.1 % formic acid) in 20 min and to 85% acetonitrile (0.1 % formic acid) in 3 min. HDMSE spectra were recorded in positive ion mode from m/z 50 to 2000 using the following settings: sampling cone of 30 V, source offset set of 60 V, source temperature of 80 °C, cone gas flow of 20 L/h, nanoflow gas pressure of 0.2 bar, and a scan time of 0.5 s. Fragmentation was triggered in the transfer region after the IMS cell using a collision energy ramp from 20 to 50 V. The doubly protonated signal of GluFib at m/z 785.8426 was acquired as lock mass.

Acquired data was processed with the ProteinLynx Global SERVER™ (PLGS) software 3.0.2 (Waters), which included Apex3D (version 2.128.9.0) and Peptide3D (version 2.115.5421.27917) as processing tools. Settings were lock mass tolerance of 0.25 Da, low energy scan of 120 counts, elevated energy scan of 30 counts, and an intensity threshold of 750 counts. The database search relied on the PLGS search engine using the following workflow parameters: Swissprot database (loaded 06/10/2015; 548,586 sequences), precursor and product MHP window (“singly protonated peptide mass of the theoretical sequence”) was set to automatic, “number by match for peptide minimum value” was 7, “number peptide for protein minimum value” was 3, “number by match for protein minimum value” was 3, “protein mass maximum Da” was 250,000, false positive rate value was 4, two missed cleavage side, trypsin as “digester reagent”, and methionine oxidation as variable modification.

**Impedance spectroscopy**

2D cell culture experiments

Prior to cell seeding the microelectrode arrays were coated by incubation with a collagen-solution (Life Technologies, Germany) (4 mg/mL in 0.02 N acetic acid) for at least one hour at 37°C. After washing the arrays with phosphate buffered saline (PBS) (Life Technologies, Germany), 1321N1 astrocytoma cells were seeded with 20,000 cells per well in 150 μL DMEM medium supplemented with 10 % FCS (Life Technologies, Germany) and cultivated at 37°C, 5 % CO_2_ until an appropriate state of confluence. For the measurement, the microelectrode arrays were placed into a self-developed multiplexer board connected to a impedance analyser (Agilent 4294A, Agilent Technologies) within a standard CO_2_-incubator one hour prior recording. Impedance spectra were automatically recorded every 20 min by applying an alternating voltage of 10 mV with frequencies ranging from 500 Hz to 5 MHz (equidistant spacing in a logarithmic scale, 11 points per decade). Instrument controlling and data recording was done with the in self-developed software IMAT Version 1.8 g. After one hour pre-monitoring, the experiment was started by the application of *A2M**. For control groups the appropriate solvent (PBS) was used. After application impedance spectra were recorded for 20h. Afterwards, cells were removed from electrodes by use of 0.25 % trypsin/EDTA solution (Life Technologies, Germany) and spectra from cell-free electrodes were recorded. The corresponding cell free spectrum was used for calculating the relative impedance values.

3D cell culture experiments

For 3D-cultures 1 x 10^6^ cells were diluted in 2 ml supplemented DMEM medium and seeded in 6-well plates for suspension cultures. The 6-well plates were incubated at 37 °C, 5 % CO_2_ on a self-developed gyratory shaker with 70 rpm for at least eight days until spheroids of appropriate size (> 150 µm) were formed. For measurements on 3D-cultures, single spheroids were selected and placed in 48-well plates with one spheroid/well. At the beginning of the experiment the spheroids were transferred to the microcavity array (MCA) which was equilibrated with PBS. The spheroids were placed into cavity with an edge length of 200 µm that is suitable for spheroids of an diameter ranging from 150-250 µm. Impedance spectra were recorded with the same measurement system that was used for 2D cultures with an adapted frequency range of 5 kHz to 5 MHz (equidistant spacing in a logarithmic scale, 11 points per decade). After measurement (0 hours) the spheroids were transferred to 48-well plates containing medium with *A2M*.* Afterwards the spheroids were kept under rotation culture (70 rpm). At discrete time points spheroids were transferred to the microcavity again for recording of impedance spectra. Microscopic images of each spheroids were taken for all time points.

Data processing and analysis

Data analysis was done with the self-developed IDAT software v3.6 which extracts the cellular contribution to the impedance magnitude spectra with the help of the spectra from cell-free electrodes. This contribution is expressed as relative impedance |Z|rel (%) = ((|Z|covered-|Z|cell-free)/|Z|cell-free x 100). Afterwards, the software determines the frequency where the relative impedance is maximum. Time traces of the relative impedance maximum were normalised to the starting point of the experiment and to the control over all to allow comparison and statistical analysis.

All statistical analysis was done using Graphpad Prism 5. All values are expressed as means± sem. Multiple group comparisons were analyzed by two-way ANOVA and Bonferroni post hoc tests. Differences between two means with P<0.05 were considered significant, P<0.01 very significant and P<0.001 extremely significant.

**Histochemistry**

Materials

Minimal essential medium (MEM), glutamine, Hanks’ Balanced Salt Solution (HBSS), normal horse serum (NHS; Gibco BRL Life Technologies, Eggenstein, Germany), glucose (Braun, Melsungen, Germany), cell-culture inserts with a pore size of 0.4 µm (Millipore, Schwalbach/Ts., Germany), vibratome (Vibratom VT 1200 S; Leica Microsystems AG, Wetzlar, Germany), six-well culture dishes (Falcon, BD Biosciences Discovery Labware, Bedford, MA, USA), streptomycin, penicillin, ascorbic acid, insulin (Boehringer, Mannheim, Germany), superfrost microscope slides from (Thermo Fisher Scientific, Rockford, IL, USA), Entellan (Merck, Darmstadt, Germany*).*

Tumor preparation

After sacrificing the animals the tumor was carefully prepared and fixed using 4 % (v/v) paraformaldehyde buffered in 0.2 M phosphate-buffer for at least 24h.

Tumor slice cultures (TSC).

Tumor slice cultures were prepared from A549 tumors by cutting in 350 µm thick slices using a vibratome at 4 °C. The slices were placed on cell-culture inserts with a pore size of 0.4 µm and transferred to six-well cell-culture dishes containing 1mL culture medium 50 % (v/v) MEM, 25 % (v/v) HBSS, 12.5 % NHS, 2 % (v/v) glutamine, 1.2 mg/mL glucose, 0.1 mg/mL streptomycin, 100 mg/mL penicillin, 0.8 mg/mL ascorbic acid and 1 mg/mL insulin; pH 7.4 per well. Culture dishes were then incubated at 35°C in a fully humidified atmosphere with 5% (v/v) CO_2_ for 24h. After one day in vitro the medium was renewed by culture medium or by culture medium containing 10nM, 30nM or 100nM *A2M**. Thereafter, the slices were incubated for two more days and fixed with 4 % (w/v) paraformaldehyde solution in 0.2 mol/L phosphate buffer for at least 12h.

Hemalaun-eosin (HE) staining and immunohistochemistry

After fixation the TSCs and tumors were embedded in paraffin and cut in 4 µm thin sections and mounted on gelatin coated microscope slides until further use. Sections were used either for hemalaun-eosin staining or for immunostaining. For HE labeling 250 µL Mayer´s hemalaun were pipetted on each slide for 5 min followed by 30 min washing with tap water. Subsequently, 250 µL eosin was added for 5 min followed by dehydration of coverslips using rising alcohols and mounting on microscope slides with Entellan. For immunohistochemistry sections were placed in methanol containing 1.5 % (v/v) of 30 % (v/v) hydrogen peroxide for 10 min to block the endogenous peroxidase. The sections were then washed three times for 10min PBS/Triton X-100, incubated with normal goat serum (1:20 in PBS/Triton X-100) for 30 min at room temperature and incubated with one of the following primary antibodies over night at 4°C: activated caspase-3, Iba1, Ki67. Subsequently, preparations were washed three times for with PBS/Triton X-100 and incubated with biotinylated goat anti-rabbit IgG (1:100) in PBS/Triton X-100 for one hour. After washing three times for 10min with PBS/Triton X-100 the slides were incubated with ExtrAvidin-Horseradish Peroxidase (1:100) in PBS/Triton X-100. Immunoreactivity was visualized by the 3.3-diamino-benzidine and slides were cover-slipped with Entellan.
