# Supplementary material for: Correction: The anti-tumorigenic activity of A2M—A lesson from the naked mole-rat
Source: PLoS One. 2018 Mar 26;13(3):e0195169. doi: 10.1371/journal.pone.0195169 (PMC5868834; doi:10.1371/journal.pone.0195169)
Supplement: S1 File — (DOCX) [file pone.0195169.s001.docx]

## Graphical Abstract


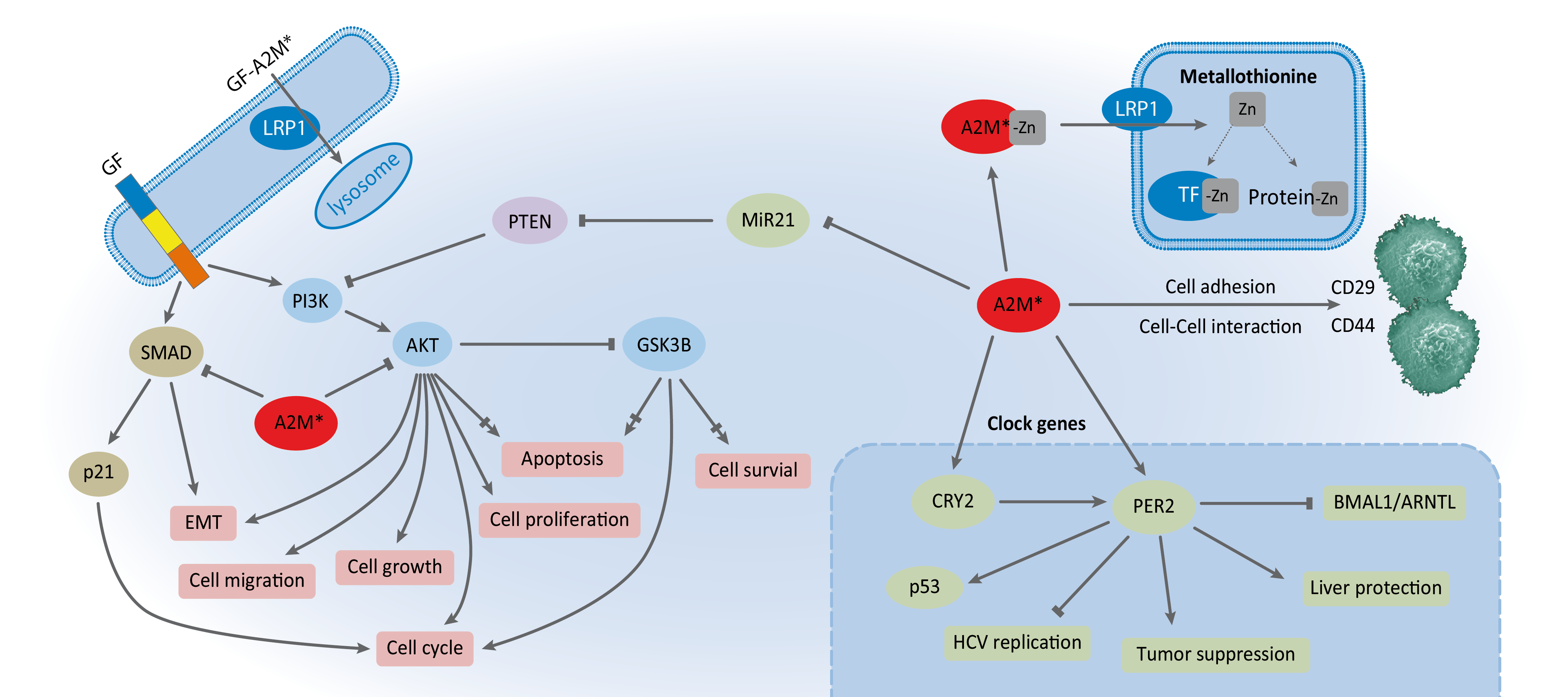


A2M is abundantly present in the blood and tissue where it senses proteolytic activities and constitutes the main carrier of Zn. Transformed A2M* binds many growth factors (GF) and mediates their clearance by receptor-mediated endocytosis via LRP1/CD91. A2M* by a yet unknown mechanism reduces the level of miR-21 known to modulate the expression of the tumour suppressor *PTEN*. Inhibition of phosphorylation of AKT, GSK3ß as well as the SMAD transcription factors (TF) has strong impact on cellular fate. The modulation of the expression of key regulators of the circadian rhythm displays a new function of A2M (
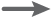
 Stimulation;
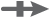
 stimulation/inhibition).
